# Tautomerism troubles: proton transfer modifies the stereochemical assignments in diastereoisomeric structures of spiro­cyclic 5-methyl-2*H*-imidazol-4-amine dimers

**DOI:** 10.1107/S205698902100668X

**Published:** 2021-11-18

**Authors:** Helen Blade, Peter N. Horton, James A. Morrison, James B. Orton, Rachel A. Sullivan, Simon J. Coles

**Affiliations:** a AstraZeneca, Oral Product Development, Pharmaceutical Technology & Development, Operations, Macclesfield, United Kingdom; b School of Chemistry, Faculty of Engineering and Physical Sciences, University of Southampton, Southampton, SO17 1BJ, United Kingdom; c AstraZeneca, Chemical Development, Pharmaceutical Technology & Development, Operations, Macclesfield, United Kingdom

**Keywords:** chirality assignment, pharmaceutical, crystal structure

## Abstract

During the racemization of a novel pharmaceutical spiro­cyclic imidazole–amine compound, namely, 6′-bromo-*N*-(6′-bromo-4-meth­oxy-4′′-methyl-3′*H*-di­spiro[cyclo­hexane-1,2′-indene-1′,2′′-imidazol]-5′′-yl)-4-meth­oxy-4′′-methyl-3′*H*-di­spiro­[cyclo­hexane-1,2′-indene-1′,2′′-imidazol]-5′′-imine, C_36_H_41_Br_2_N_5_O_2_, two impurities were isolated. These impurities were clearly dimers from mass spectroscopic analysis, however single-crystal diffraction characterization was required for the assignment of stereochemistry. The single-crystal diffraction results revealed subtly different structures to those proposed, due to an unexpected proton transfer. The dimers contain four stereocentres, but two of primary inter­est, and are centrosymmetric, so after careful structure refinement and close inspection it was possible to unambiguously assign the stereochemistry of both the homochiral [(*S*),(*S*)- and (*R*),(*R*)-] and the heterochiral [(*S*),(*R*)- and (*R*),(*S*)-] compounds.

## Abstract 2

Single-crystal structure analysis was required to correctly identify the mol­ecular structure and stereochemistry assignment of unexpected impurities in the preparation of a novel pharmaceutical spiro­cyclic imidazole-amine compound.

## Chemical context

**Figure 1:**
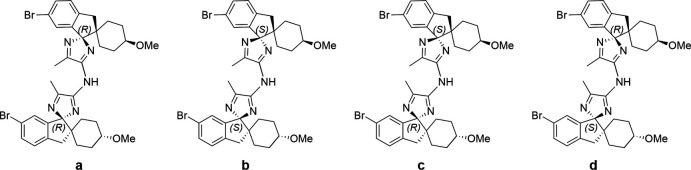
Proposed structures of the dimeric impurities, comprising the (*R*),(*R*)-, (*S*),(*S*)-, (*R*),(*S*)- and (*S*),(*R*)- compounds, respectively.

**Figure 2:**
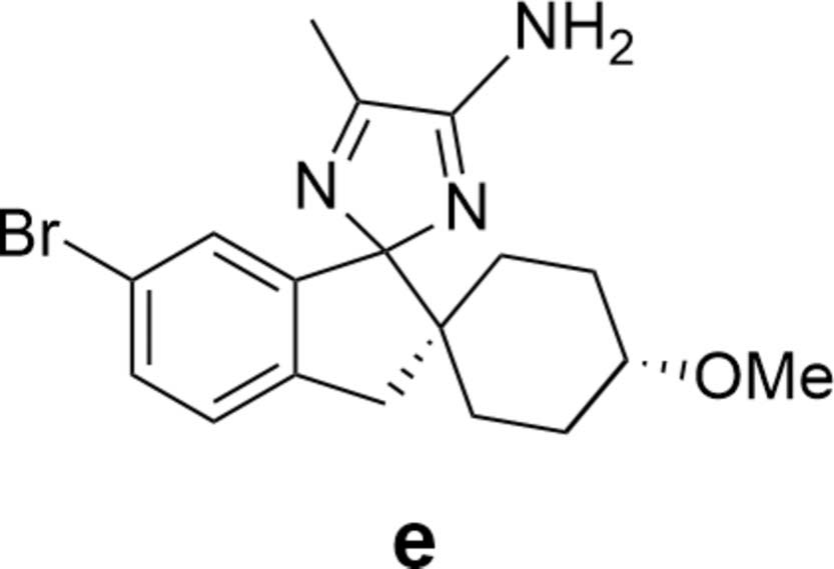
5-methyl-2H-imidazol-4-amine, compound **e**.

During the racemization of an enanti­opure spiro­cyclic 5-methyl-2*H*-imidazol-4-amine, two impurities were observed by reverse phase-HPLC, which were subsequently rationalized as a combination of the homochiral compounds (*S*),(*S*)-(*R*),(*R*)-, and heterochiral compounds (*S*),(*R*)- and (*R*),(*S*)*-* (see Fig. 1 ▸ for the proposed 2D structures). Solution-state NMR and mass spectrometry analysis revealed that these impurities were dimers of the 5-methyl-2*H*-imidazol-4-amine compound **e** (see Fig. 2 ▸); no diagnostic signals were observed in the solution-state NMR and therefore single-crystal structure determination was required to allow assignment of the absolute configuration of the impurities observed.

The chemical shifts from the solution-state NMR are given in Section 6 below. As related enanti­omers are indistinguishable by solution-state NMR, single crystal X-ray diffraction analysis was sought to enable an unambiguous assignment, revealing structures **1** and **2**. This analysis not only enabled the identification of the correct absolute structure, but also revealed that there was, in fact, a subtle variation to the proposed structures. Crystal structures were obtained for both the impurities observed, which revealed that the homochiral and heterochiral structures differed from those proposed (**a**, **b**, **c** and **d**) due to hydrogen migration from the bridging nitro­gen centre to the closest imidazole group. 
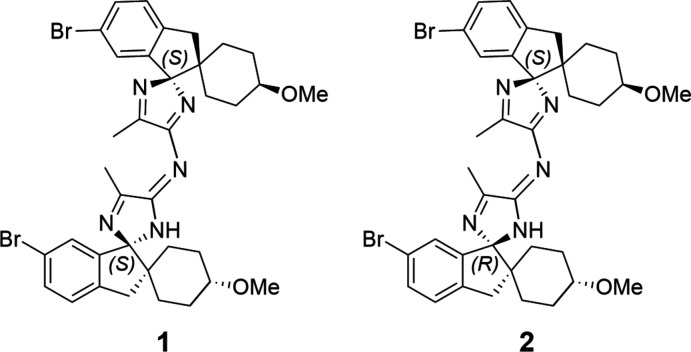


## Structural commentary

**Figure 3:**
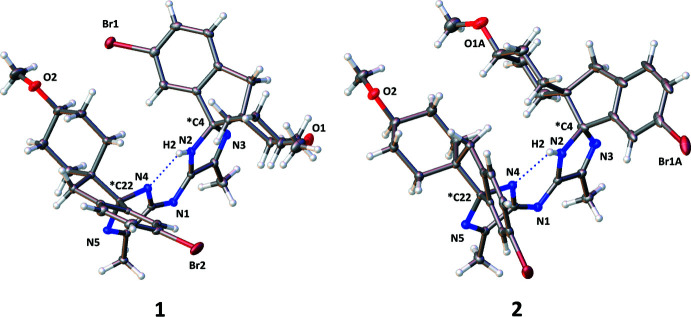
The mol­ecular structures with atomic numbering schemes (non-carbon and hydrogen atoms only for clarity) for **1** and **2** respectively. The chiral centres are marked with an asterisk and for clarity only the enanti­omer solved in the asymmetric unit of each structure is shown.

Both structures solved and refined satisfactorily in the centrosymmetric space group *P*2_1_/*n*. Therefore, both possible diastereoisomers, *RR*/*SS* (in structure **1**) and *RS*/*SR* (in structure **2**), are present in equal amounts in their respective crystal. The structures along with their atomic numbering schemes are illustrated in Fig. 3 ▸.

**Figure 4:**
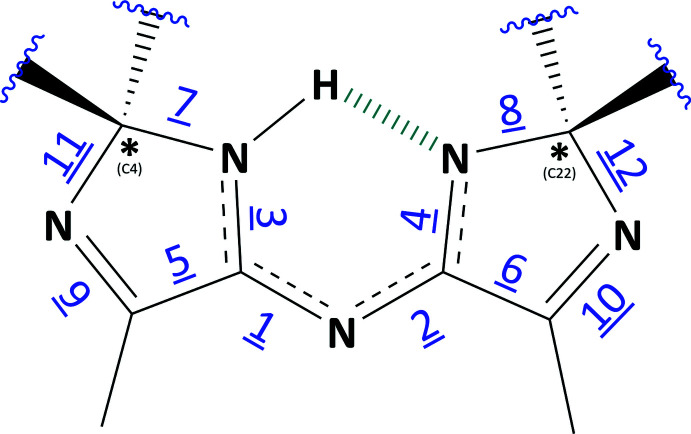
Labelled bonds in the N-bridged bis-imidazole core, with associated bond lengths for **1** and **2** denoted in Table 1 ▸.

Fig. 3 ▸ shows that in both cases an unexpected proton transfer from the bridging amine centre (N1 in both structures) to the spiro-imidazole nitro­gen (N2 in both structures) had occurred. Examination of residual electron density maps (see *Refinement* section and Fig. 6 ▸) of both structures, supported by inter­preting the bond lengths around these nitro­gen centres, confirmed the location of the hydrogen atom and therefore the fact that this migration has occurred. It was, however, necessary to restrain the N2—H2 bond in structure **1**, otherwise it refined to a value slightly shorter than expected. This transfer results in a perturbation of the bonding pattern within the imidazole rings for both structures. The bond conjugation between these rings is extended *via* the bridging nitro­gen (N1), which makes the formal nature of the double and single bonds in these ring systems less clear, as depicted in Fig. 4 ▸ and Table 1 ▸.

This perturbation of bonding means particularly close attention must be paid to the formal chirality assignment of the stereocentres C4 and C22 in both structures **1** and **2**, as it is dependent on analysis of the surrounding imidazole bond lengths. The definitive Cahn–Ingold–Prelog assignment (Cahn *et al.*, 1966 ▸) of these stereocentres required a manual approach as the algorithms in both *PLATON* (Spek, 2020 ▸) and *Mercury* (Macrae *et al.*, 2020 ▸) software gave inaccurate results, due to the ambiguity of bond order altering the priority of the bonds connected to the stereocentres. In both structures, the distances between atoms C1 and N2 (**1**) and C19 and N4 (**2**) have more double-bond character than single. This results in a formal designation of *RR* (and *SS*) and *RS* (and *SR*) in the refined structures of **1** and **2**, respectively.

The hydrogen-atom location also results in the formation of a strong intra­molecular hydrogen bond between both imidazole rings, mediated by a N2—H2⋯N4 inter­action in each structure (Tables 2 ▸ and 3 ▸).

## Supra­molecular features

**Figure 5:**
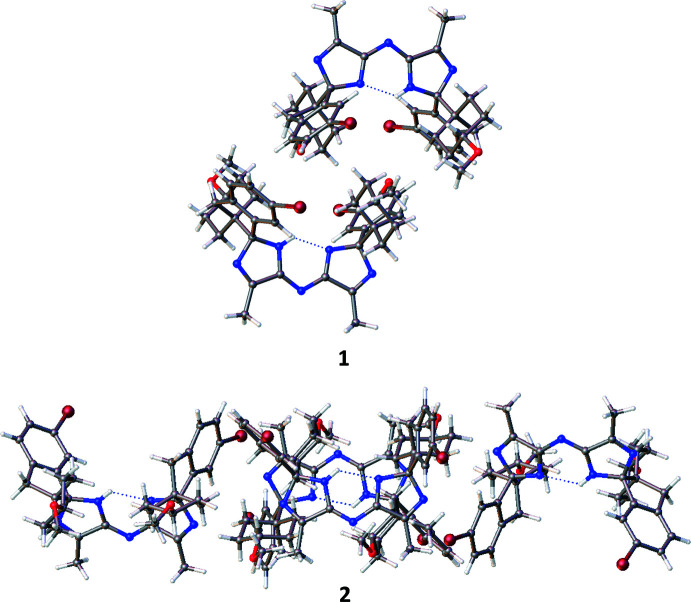
The predominant packing motifs in the structures of **1** and **2**.

The packing arrangement for both structures **1** and **2** are shown in Fig. 5 ▸. In both compounds there are no hydrogen-bonding inter­actions present within the structure, other than that of the N2—H2⋯N4 intra­molecular hydrogen bond. This intra­molecular inter­action is an additional factor influencing the delocalization of bonding in these ring systems.

The packing is likely to be dominated by dispersive inter­actions and the differences between the two motifs will be small. These mol­ecules have a ridge-tile shape and the structure of **1** involves insertion of a sidewall of one mol­ecule into the cleft of another; this motif contains some small voids with a volume of approximately 30 Å^2^ calculated using *Mercury* (Macrae *et al.*, 2020 ▸). However, mol­ecules in the structure of **2** assemble in a side-by-side manner into a strand, which allows complementary head-to-tail stacking of strands and is more packing efficient (using the same settings in *Mercury* no voids are calculated).

## Database survey

These mol­ecular structures are relatively unique in solid-state chemistry. A search of the CSD (CSD version 5.42, updates of Feb 2021; Groom *et al.*, 2016 ▸) yielded no results for structures with a similarity to the overall mol­ecule or to the motif of the methyl-imidazole-amine bridged dimer. There were also no structures found for a spiro group with similar substituents.

2*H*-Imidazoles are well known in organic chemistry and a name search for these in the CSD revealed 677 structures. A combination of this search with that of the substructure of the imidazole core of this system, where the bonds are considered to be ‘double’ produces 50 hits, while there are no results if these bonds are defined as ‘delocalized’. Analysis of the hit lists does not reveal any structures similar to those reported herein and therefore does not provide any insight as to how the bonding should be assigned.

## Synthesis and crystallization

Solid samples of **1** and **2** were isolated from a reaction to form a spiro­cyclic 5-methyl-2*H*-imidazol-4-amine, during which they were formed as an impurity product and were subsequently isolated. Single crystals of compounds **1** and **2** were grown by slow evaporation at room temperature from individual solutions of ethyl acetate (200 mL g^−1^). Each mixture was allowed to evaporate to dryness over the period of a week. Both compounds formed colourless block-shaped crystals.

## Characterization by spectroscopic techniques

The following NMR and mass spectrometry data were collected.

Compound **a**, (*R*,*R*)/(*S*,*S*)-(1*r*,1′*S*,4*S*,*E*)-6′-bromo-*N*-[(1*r*,1′*S*,4*S*)-6′-bromo-4-meth­oxy-4′′-methyl-3′*H*-di­spiro­[cyclo­hexane-1,2′-indene-1′,2′′-imidazol-5′′-yl]-4-meth­oxy-4′′-methyl-3′*H*-di­spiro­[cyclo­hexane-1,2′-indene-1′,2′′-imidazol]-5′′-imine (and enanti­omer):

^1^H NMR (500 MHz, CDCl_3_) 1.06 (*td*, *J* = 13.7, 3.8 Hz, 1H), 1.22–1.47 (*m*, 3H), 1.65 (*dd*, *J* = 12.8, 2.9 Hz, 1H), 1.68–1.77 (*m*, 1H), 1.85–1.94 (*m*, 1H), 1.94–2.03 (*m*, 1H), 2.41 (*s*, 3H), 2.91–3.01 (*m*, 1H), 3.06–3.17 (*m*, 2H), 3.31 (*s*, 3H), 6.90 (*d*, *J* = 1.7 Hz, 1H), 7.19 (*d*, *J* = 8.0 Hz, 1H), 7.38 (*dd*, *J* = 8.0, 1.9 Hz, 1H). ^13^C NMR (126 MHz, CDCl_3_) 14.11, 28.26, 28.39, 28.98, 30.46, 39.23, 53.08, 53.14, 55.43, 79.02, 104.96, 120.06, 125.61, 127.16, 131.63, 141.17, 142.20, 165.37, 165.82. LC–MS (ESI, *M* + H^+^) 734.2, 736.1, 738.1

Compound **c**, (*R*,*S*)/(*S*,*R*)-(1*r*,1′*S*,4*S*,*E*)-6′-bromo-*N*-[(1*r*,1′*S*,4*S*)-6′-bromo-4-meth­oxy-4′′-methyl-3′*H*-di­spiro­[cyclo­hexane-1,2′-indene-1′,2′′-imidazol-5′′-yl]-4-meth­oxy-4′′-methyl-3′*H*-di­spiro­[cyclo­hexane-1,2′-indene-1′,2′′-imidazol]-5′′-imine (and enanti­omer):

^1^H NMR (500 MHz, CDCl_3_) 1.06 (*td*, *J* = 13.6, 3.7 Hz, 1H), 1.20 (*td*, *J* = 13.3, 3.5 Hz, 1H), 1.31 (*dqt*, *J* = 21.3, 8.2, 4.1 Hz, 2H), 1.5–1.6 (*m*, 1H), 1.65–1.78 (m, 1H), 1.85–2.01 (*m*, 2H), 2.41 (*s*, 3H), 3.03 (*td*, *J* = 10.7, 5.3 Hz, 1H), 3.13 (*s*, 2H), 3.32 (*s*, 3H), 6.86 (*d*, *J* = 1.8 Hz, 1H), 7.20 (*d*, *J* = 8.0 Hz, 1H), 7.38 (*dd*, *J* = 8.0, 1.9 Hz, 1H). ^13^C NMR (126 MHz, CDCl_3_) 14.11, 28.05, 28.32, 28.88, 29.94, 39.28, 53.05, 55.47, 78.49, 104.94, 119.80, 125.04, 127.27, 131.49, 141.48, 142.80, 165.56, 165.73. LC–MS (ESI, *M* + H^+^) 734.2, 736.1, 738.1

## Refinement

**Figure 6:**
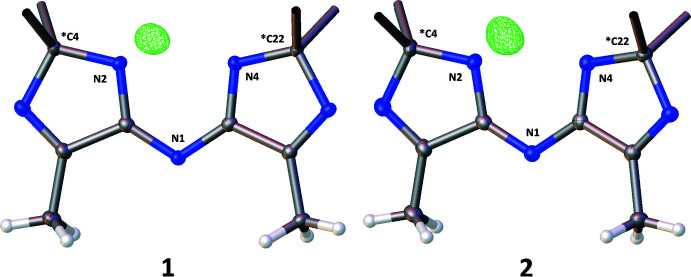
The 3D residual electron difference maps from the refinements of **1** and **2**. The green wireframe is drawn at a threshold of >0.4 electrons /Å^−3^.

The crystal data, data collection and refinement details for structures **1** and **2** are summarized in Table 4 ▸ and were obtained by following a previously published approach (Coles & Gale, 2012 ▸). Further details of these experiments are given at the end of this section. The structure refinements of both **1** and **2** demonstrated that the hydrogen atom anti­cipated to be bound to the bridging nitro­gen (labelled N1 in both structures) was in fact bound to one of the adjacent imidazole nitro­gen atoms (labelled N2 in both structures). This was confirmed by inspection of residual electron difference maps. Fig. 6 ▸ depicts 3D representations of the residual electron difference map around the bis-imidazole cores of **1** and **2**, the green wireframes are drawn at a threshold of >0.4 electrons/Å^−3^ and highlight the location of the hydrogen atoms.

The structures of **1** and **2** solved in the space group *P*2_1_/*n* (# 14) using dual methods in the *SHELXT* (Sheldrick, 2015*a* ▸) structure-solution program and refined by full-matrix least-squares minimization on *F^2^* using *SHELXL2018/3* (Sheldrick, 2015*b* ▸). All non-hydrogen atoms were refined anisotropically. The position of the N—H atom H2 was located from the difference map and refined with its thermal parameter linked to that of its parent atom, N2. The positions of the remaining C—H atoms were calculated geometrically and refined using a riding model.

**Figure 7:**
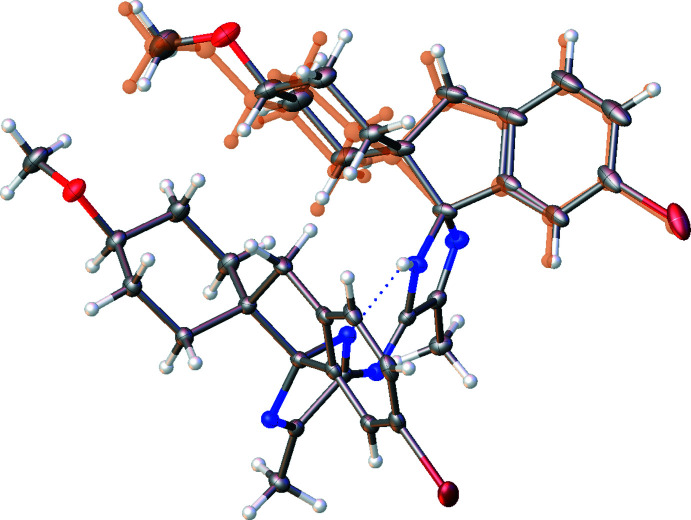
The disorder modelling in structure **2**, with displacement ellipsoids drawn at the 50% probability level and the minor component highlighted in orange (10.8%).

The disordered atoms of **2** (Br1*A*/Br1*B*, O1*A*/O1*B* and C5*A*/C5*B* > C18*A*/C18*B*), have been modelled over two positions using geometric parameter restraints. In addition, the geometry of the minor benzene ring (C5*B* > C10*B*) was constrained to be a regular hexa­gon with bond lengths of 1.39 Å. All minor atomic positions were modelled isotropically with the thermal parameters of atoms Br1*A* and Br1*B* restrained and those of atoms O1*B* and C5*B* > C18*B*, constrained to be the same. Applying the above to the refinement conserved realistic chemical geometries and lowered the *R*_1_ value from 2.74% to 2.20%. Fig. 7 ▸ depicts the disorder modelling in structure **2**, with displacement ellipsoids drawn at the 50% probability level and the minor component highlighted in orange (10.8%).

## Supplementary Material

Crystal structure: contains datablock(s) 2, 1, New_Global_Publ_Block. DOI: 10.1107/S205698902100668X/dj2028sup1.cif

Structure factors: contains datablock(s) 1. DOI: 10.1107/S205698902100668X/dj20281sup2.hkl

Structure factors: contains datablock(s) 2. DOI: 10.1107/S205698902100668X/dj20282sup3.hkl

CCDC references: 2092542, 2092541

Additional supporting information:  crystallographic information; 3D view; checkCIF report

## Figures and Tables

**Table 1:** Bond lengths (Å) in the N-bridged bis-imidazole core for structures **1** and **2**, with bonds denoted as in Fig. 4 ▸

Bond	Structure **1**	Bond order	Structure **2**	Bond order
1 (N1⋯C1)	**1.3327 (19)**	**delocalized**	**1.3081 (18)**	**delocalized**
2 (N1⋯C19)	**1.3616 (18)**	**delocalized**	**1.3853 (18)**	**delocalized**
3 (C1⋯N2)	**1.3250 (19)**	**delocalized**	**1.3374 (18)**	**delocalized**
4 (C19⋯N4)	**1.3115 (18)**	**delocalized**	**1.2983 (18)**	**delocalized**
5 (C1—C2)	1.4940 (19)	single	1.4959 (19)	single
6 (C19—C20)	1.4925 (19)	single	1.4940 (18)	single
7 (N2—C4)	1.4471 (18)	single	1.4509 (17)	single
8 (N4—C22)	1.4513 (17)	single	1.4791 (17)	single
9 (C2—N3)	1.2818 (19)	double	1.2800 (19)	double
10 (N5—C20)	1.2809 (19)	double	1.2837 (18)	double
11 (N3—C4)	1.4770 (17)	single	1.4759 (18)	single
12 (N5—C22)	1.4816 (17)	single	1.4635 (17)	single

**Table 2:** Hydrogen-bond geometry (Å, °) for **1** [Chem scheme1]

*D*—H⋯*A*	*D*—H	H⋯*A*	*D*⋯*A*	*D*—H⋯*A*
N2—H2⋯N4	0.826 (19)	2.064 (18)	2.6456 (16)	127.2 (15)

**Table 3:** Hydrogen-bond geometry (Å, °) for **2** [Chem scheme1]

*D*—H⋯*A*	*D*—H	H⋯*A*	*D*⋯*A*	*D*—H⋯*A*
N2—H2⋯N4	0.82 (1)	1.95 (2)	2.5549 (16)	129 (2)

**Table 4:** Experimental details

	**1**	**2**
Crystal data		
Chemical formula	C_36_H_41_Br_2_N_5_O_2_	C_36_H_41_Br_2_N_5_O_2_
*M*_r_	735.56	735.56
Crystal system, space group	Monoclinic, *P*2_1_/*n*	Monoclinic, *P*2_1_/*n*
Temperature (K)	100	100
*a*, *b*, *c* (Å)	10.18956 (5), 13.92084 (5), 25.48643 (14)	13.19297 (6), 17.60010 (8), 15.25349 (8)
β (°)	113.5742 (7)	104.4018 (5)
*V* (Å^3^)	3313.47 (3)	3430.52 (3)
*Z*	4	4
Radiation type	Cu *K*α	Cu *K*α
μ (mm^−1^)	3.42	3.30
Crystal size (mm)	0.18 × 0.05 × 0.03	0.31 × 0.07 × 0.05

Data collection		
Diffractometer	Rigaku 007HF equipped with Varimax confocal mirrors and an AFC11 goniometer and HyPix 6000 detector	Rigaku 007HF equipped with Varimax confocal mirrors and an AFC11 goniometer and HyPix 6000 detector
Absorption correction	Gaussian (*CrysAlis PRO*; Rigaku OD, 2019 ▸)	Gaussian (*CrysAlis PRO*; Rigaku OD, 2019 ▸)
*T*_min_, *T*_max_	0.621, 1.000	0.654, 1.000
No. of measured, independent and observed [*I* > 2σ(*I*)] reflections	118177, 6068, 5967	97773, 6285, 6257
*R*_int_	0.031	0.023
(sin θ/λ)_max_ (Å^−1^)	0.602	0.602

Refinement		
*R*[*F* ^2^ > 2σ(*F* ^2^)], *wR*(*F* ^2^), *S*	0.022, 0.056, 1.06	0.024, 0.057, 1.06
No. of reflections	6068	6285
No. of parameters	454	413
No. of restraints	15	1
H-atom treatment	H atoms treated by a mixture of independent and constrained refinement	H atoms treated by a mixture of independent and constrained refinement
Δρ_max_, Δρ_min_ (e Å^−3^)	0.33, −0.39	0.33, −0.44

Computer programs: *CrysAlis PRO* (Rigaku OD, 2019 ▸), *SHELXT* (Sheldrick, 2015*a* ▸), *SHELXL2018/3* (Sheldrick, 2015*b* ▸) and *OLEX2* (Dolomanov *et al.*, 2009 ▸).

## supplementary crystallographic information

### (R,R)/(S,S)-(1r,1'S,4S,E)-6'-Bromo-N-[(1r,1'S,4S)-6'-bromo-4-methoxy-4''-methyl-3'H-dispiro[cyclohexane-1,2'-indene-1',2''-imidazol-5''-yl]-4-methoxy-4''-methyl-3'H-dispiro[cyclohexane-1,2'-indene-1',2''-imidazol]-5''-imine (2) Crystal data

**Table:**

C_36_H_41_Br_2_N_5_O_2_	*F*(000) = 1512
*M**_r_* = 735.56	*D*_x_ = 1.474 Mg m^−^^3^
Monoclinic, *P*2_1_/*n*	Cu *K*α radiation, λ = 1.54178 Å
*a* = 10.18956 (5) Å	Cell parameters from 82488 reflections
*b* = 13.92084 (5) Å	θ = 3.2–70.3°
*c* = 25.48643 (14) Å	µ = 3.42 mm^−^^1^
β = 113.5742 (7)°	*T* = 100 K
*V* = 3313.47 (3) Å^3^	Block, colourless
*Z* = 4	0.18 × 0.05 × 0.03 mm

### (R,R)/(S,S)-(1r,1'S,4S,E)-6'-Bromo-N-[(1r,1'S,4S)-6'-bromo-4-methoxy-4''-methyl-3'H-dispiro[cyclohexane-1,2'-indene-1',2''-imidazol-5''-yl]-4-methoxy-4''-methyl-3'H-dispiro[cyclohexane-1,2'-indene-1',2''-imidazol]-5''-imine (2) Data collection

**Table:**

Rigaku 007HF equipped with Varimax confocal mirrors and an AFC11 goniometer and HyPix 6000 detector diffractometer	6068 independent reflections
Radiation source: Rotating anode, Rigaku 007 HF	5967 reflections with *I* > 2σ(*I*)
Mirror monochromator	*R*_int_ = 0.031
Detector resolution: 10 pixels mm^-1^	θ_max_ = 68.2°, θ_min_ = 3.7°
profile data from ω–scans	*h* = −12→12
Absorption correction: gaussian (CrysAlisPro; Rigaku OD, 2019)	*k* = −16→16
*T*_min_ = 0.621, *T*_max_ = 1.000	*l* = −30→30
118177 measured reflections	

### (R,R)/(S,S)-(1r,1'S,4S,E)-6'-Bromo-N-[(1r,1'S,4S)-6'-bromo-4-methoxy-4''-methyl-3'H-dispiro[cyclohexane-1,2'-indene-1',2''-imidazol-5''-yl]-4-methoxy-4''-methyl-3'H-dispiro[cyclohexane-1,2'-indene-1',2''-imidazol]-5''-imine (2) Refinement

**Table:**

Refinement on *F*^2^	Hydrogen site location: mixed
Least-squares matrix: full	H atoms treated by a mixture of independent and constrained refinement
*R*[*F*^2^ > 2σ(*F*^2^)] = 0.022	*w* = 1/[σ^2^(*F*_o_^2^) + (0.0259*P*)^2^ + 2.2368*P*] where *P* = (*F*_o_^2^ + 2*F*_c_^2^)/3
*wR*(*F*^2^) = 0.056	(Δ/σ)_max_ = 0.001
*S* = 1.06	Δρ_max_ = 0.33 e Å^−^^3^
6068 reflections	Δρ_min_ = −0.39 e Å^−^^3^
454 parameters	Extinction correction: SHELXL-2018/3 (Sheldrick 2015b), Fc^*^=kFc[1+0.001xFc^2^λ^3^/sin(2θ)]^-1/4^
15 restraints	Extinction coefficient: 0.00013 (2)
Primary atom site location: dual	

### (R,R)/(S,S)-(1r,1'S,4S,E)-6'-Bromo-N-[(1r,1'S,4S)-6'-bromo-4-methoxy-4''-methyl-3'H-dispiro[cyclohexane-1,2'-indene-1',2''-imidazol-5''-yl]-4-methoxy-4''-methyl-3'H-dispiro[cyclohexane-1,2'-indene-1',2''-imidazol]-5''-imine (2) Special details

**Table:**

Geometry. All esds (except the esd in the dihedral angle between two l.s. planes) are estimated using the full covariance matrix. The cell esds are taken into account individually in the estimation of esds in distances, angles and torsion angles; correlations between esds in cell parameters are only used when they are defined by crystal symmetry. An approximate (isotropic) treatment of cell esds is used for estimating esds involving l.s. planes.
Refinement. This diastereoisomer has crystallised in the centrosymmetric space group P2_1_/n; meaning that both RS and SR forms of the API must be present in equal amounts within the crystal. The atoms of both methoxy groups (O1, C18 and O2, C36), have been modelled as single sites with large thermal ellipsoids. This was found to be the most appropriate model; however, these large ellipsoids result in two checkCIF C-alerts. The N2-H2 bond length has been restrained, otherwise it refined to an unrealistic value.

### (R,R)/(S,S)-(1r,1'S,4S,E)-6'-Bromo-N-[(1r,1'S,4S)-6'-bromo-4-methoxy-4''-methyl-3'H-dispiro[cyclohexane-1,2'-indene-1',2''-imidazol-5''-yl]-4-methoxy-4''-methyl-3'H-dispiro[cyclohexane-1,2'-indene-1',2''-imidazol]-5''-imine (2) Fractional atomic coordinates and isotropic or equivalent isotropic displacement parameters (Å^2^)

**Table:**

	*x*	*y*	*z*	*U*_iso_*/*U*_eq_	Occ. (<1)
Br1A	0.94977 (8)	−0.13936 (5)	0.43736 (3)	0.0374 (2)	0.892 (3)
Br1B	0.9408 (7)	−0.1378 (3)	0.4382 (2)	0.0172 (9)*	0.108 (3)
Br2	1.07263 (2)	−0.11819 (2)	0.78926 (2)	0.02647 (6)	
C1	0.53448 (14)	0.13684 (9)	0.50383 (6)	0.0145 (3)	
C2	0.43133 (15)	0.12704 (10)	0.44275 (6)	0.0165 (3)	
C3	0.27976 (15)	0.09625 (11)	0.42505 (6)	0.0202 (3)	
H3A	0.229686	0.101766	0.383384	0.030*	
H3B	0.232780	0.137362	0.443573	0.030*	
H3C	0.277007	0.029346	0.436529	0.030*	
C4	0.63956 (15)	0.17633 (11)	0.44190 (6)	0.0193 (3)	
C5A	0.7365 (5)	0.1160 (3)	0.4219 (2)	0.0222 (4)	0.892 (3)
C5B	0.726 (5)	0.1225 (19)	0.4235 (19)	0.0203 (18)*	0.108 (3)
C6B	0.779 (5)	0.0303 (19)	0.4395 (18)	0.0203 (18)*	0.108 (3)
H6B	0.758545	−0.003496	0.467690	0.024*	0.108 (3)
C7B	0.862 (4)	−0.0124 (15)	0.4141 (16)	0.0203 (18)*	0.108 (3)
C8B	0.892 (3)	0.0371 (14)	0.3728 (13)	0.0203 (18)*	0.108 (3)
H8B	0.949075	0.007835	0.355409	0.024*	0.108 (3)
C9B	0.839 (4)	0.1293 (14)	0.3568 (14)	0.0203 (18)*	0.108 (3)
H9B	0.859224	0.163089	0.328615	0.024*	0.108 (3)
C10B	0.756 (5)	0.1720 (15)	0.3822 (17)	0.0203 (18)*	0.108 (3)
C6A	0.7881 (6)	0.0251 (3)	0.4394 (2)	0.0232 (4)	0.892 (3)
H6A	0.764777	−0.009365	0.466683	0.028*	0.892 (3)
C7A	0.8764 (5)	−0.0138 (2)	0.41507 (19)	0.0295 (6)	0.892 (3)
C8A	0.9095 (4)	0.0346 (3)	0.37454 (17)	0.0378 (7)	0.892 (3)
H8A	0.969356	0.005467	0.358518	0.045*	0.892 (3)
C9A	0.8551 (5)	0.1254 (3)	0.35756 (19)	0.0400 (8)	0.892 (3)
H9A	0.877176	0.159069	0.329666	0.048*	0.892 (3)
C10A	0.7676 (6)	0.1679 (2)	0.3814 (2)	0.0308 (6)	0.892 (3)
C11A	0.6999 (8)	0.2652 (3)	0.3726 (2)	0.0333 (7)	0.892 (3)
H11A	0.608818	0.266382	0.338153	0.040*	0.892 (3)
H11B	0.764944	0.314851	0.368599	0.040*	0.892 (3)
C11B	0.697 (6)	0.273 (2)	0.3769 (19)	0.0203 (18)*	0.108 (3)
H11C	0.613084	0.281278	0.340010	0.024*	0.108 (3)
H11D	0.770694	0.320734	0.378786	0.024*	0.108 (3)
C12A	0.6733 (3)	0.28113 (17)	0.42800 (14)	0.0223 (6)	0.892 (3)
C12B	0.651 (3)	0.2870 (12)	0.4280 (12)	0.0203 (18)*	0.108 (3)
C13A	0.5515 (2)	0.3498 (3)	0.42170 (15)	0.0284 (6)	0.892 (3)
H13A	0.528888	0.344473	0.455909	0.034*	0.892 (3)
H13B	0.465152	0.330764	0.387914	0.034*	0.892 (3)
C13B	0.518 (3)	0.349 (2)	0.4150 (15)	0.0203 (18)*	0.108 (3)
H13C	0.483509	0.338398	0.445655	0.024*	0.108 (3)
H13D	0.442776	0.326030	0.378784	0.024*	0.108 (3)
C14A	0.5885 (2)	0.45441 (16)	0.41473 (9)	0.0307 (5)	0.892 (3)
H14A	0.603163	0.461269	0.378796	0.037*	0.892 (3)
H14B	0.507726	0.496456	0.412131	0.037*	0.892 (3)
C14B	0.535 (2)	0.4608 (14)	0.4093 (8)	0.0203 (18)*	0.108 (3)
H14C	0.548316	0.474167	0.373635	0.024*	0.108 (3)
H14D	0.445952	0.493368	0.406538	0.024*	0.108 (3)
C15A	0.7235 (3)	0.48558 (15)	0.46518 (8)	0.0267 (4)	0.892 (3)
H15A	0.706406	0.480777	0.501104	0.032*	0.892 (3)
C15B	0.654 (2)	0.4996 (12)	0.4572 (7)	0.0203 (18)*	0.108 (3)
H15B	0.635274	0.492084	0.492578	0.024*	0.108 (3)
C16A	0.8469 (2)	0.42084 (16)	0.47028 (8)	0.0261 (4)	0.892 (3)
H16A	0.933141	0.440372	0.503969	0.031*	0.892 (3)
H16B	0.867971	0.428206	0.435805	0.031*	0.892 (3)
C16B	0.791 (3)	0.4494 (14)	0.4659 (7)	0.0203 (18)*	0.108 (3)
H16C	0.868915	0.476239	0.500163	0.024*	0.108 (3)
H16D	0.814573	0.461434	0.432469	0.024*	0.108 (3)
C17A	0.8131 (2)	0.31553 (15)	0.47662 (9)	0.0215 (5)	0.892 (3)
H17A	0.804927	0.306878	0.513749	0.026*	0.892 (3)
H17B	0.893491	0.275115	0.477054	0.026*	0.892 (3)
C17B	0.781 (2)	0.3407 (15)	0.4737 (10)	0.0203 (18)*	0.108 (3)
H17C	0.779365	0.329361	0.511730	0.024*	0.108 (3)
H17D	0.870363	0.311012	0.474303	0.024*	0.108 (3)
C18A	0.6803 (3)	0.65329 (16)	0.46879 (12)	0.0413 (6)	0.892 (3)
H18A	0.583914	0.650527	0.438231	0.062*	0.892 (3)
H18B	0.722462	0.716483	0.468597	0.062*	0.892 (3)
H18C	0.674585	0.642820	0.505843	0.062*	0.892 (3)
C18B	0.7490 (18)	0.6576 (11)	0.4964 (7)	0.0203 (18)*	0.108 (3)
H18D	0.754713	0.724310	0.485220	0.030*	0.108 (3)
H18E	0.845719	0.630669	0.514422	0.030*	0.108 (3)
H18F	0.702936	0.655535	0.523542	0.030*	0.108 (3)
C19	0.60745 (14)	0.15389 (9)	0.60063 (6)	0.0138 (3)	
C20	0.57460 (14)	0.16738 (9)	0.65227 (6)	0.0144 (3)	
C21	0.43197 (15)	0.15223 (11)	0.65370 (6)	0.0188 (3)	
H21A	0.398441	0.087093	0.640470	0.028*	
H21B	0.363969	0.199100	0.628608	0.028*	
H21C	0.439273	0.160477	0.692959	0.028*	
C22	0.80075 (14)	0.20290 (10)	0.67386 (6)	0.0144 (3)	
C23	0.93006 (14)	0.14103 (10)	0.70748 (6)	0.0152 (3)	
C24	0.93173 (15)	0.05021 (10)	0.72998 (6)	0.0170 (3)	
H24	0.845787	0.018643	0.726256	0.020*	
C25	1.06528 (16)	0.00721 (11)	0.75835 (6)	0.0195 (3)	
C26	1.19161 (16)	0.05226 (11)	0.76421 (6)	0.0218 (3)	
H26	1.280946	0.021263	0.784060	0.026*	
C27	1.18686 (15)	0.14333 (11)	0.74081 (6)	0.0201 (3)	
H27	1.272763	0.174655	0.744257	0.024*	
C28	1.05525 (15)	0.18787 (10)	0.71239 (6)	0.0173 (3)	
C29	1.02117 (15)	0.28508 (10)	0.68463 (6)	0.0189 (3)	
H29A	1.022639	0.283712	0.646033	0.023*	
H29B	1.090366	0.333869	0.708176	0.023*	
C30	0.86808 (14)	0.30664 (10)	0.68100 (6)	0.0158 (3)	
C31	0.78163 (17)	0.37358 (10)	0.63154 (6)	0.0215 (3)	
H31A	0.682777	0.379223	0.629414	0.026*	
H31B	0.776406	0.345604	0.595042	0.026*	
C32	0.84940 (17)	0.47367 (11)	0.63930 (6)	0.0237 (3)	
H32A	0.945356	0.468505	0.638376	0.028*	
H32B	0.789723	0.515270	0.607076	0.028*	
C33	0.86370 (16)	0.51995 (10)	0.69566 (6)	0.0199 (3)	
H33	0.766053	0.534957	0.693902	0.024*	
C34	0.94019 (15)	0.45383 (10)	0.74631 (6)	0.0184 (3)	
H34A	0.935315	0.482069	0.781137	0.022*	
H34B	1.042379	0.449010	0.752665	0.022*	
C35	0.87464 (15)	0.35300 (10)	0.73697 (6)	0.0166 (3)	
H35A	0.776587	0.356845	0.735909	0.020*	
H35B	0.932251	0.311634	0.769691	0.020*	
C36	0.86287 (19)	0.68719 (11)	0.67521 (7)	0.0294 (4)	
H36A	0.785107	0.699087	0.687764	0.044*	
H36B	0.822439	0.673693	0.634040	0.044*	
H36C	0.924525	0.744030	0.683035	0.044*	
N1	0.50182 (12)	0.12806 (8)	0.54828 (5)	0.0145 (2)	
N2	0.65823 (13)	0.16116 (9)	0.50079 (5)	0.0158 (2)	
H2	0.730 (2)	0.1702 (12)	0.5305 (8)	0.019*	
N3	0.48867 (13)	0.15027 (9)	0.40810 (5)	0.0197 (3)	
N4	0.74130 (12)	0.17302 (8)	0.61310 (5)	0.0144 (2)	
N5	0.68690 (12)	0.19542 (8)	0.69490 (5)	0.0147 (2)	
O1A	0.76650 (15)	0.58132 (9)	0.45969 (6)	0.0329 (4)	0.892 (3)
O1B	0.6675 (12)	0.6029 (8)	0.4471 (4)	0.0203 (18)*	0.108 (3)
O2	0.94500 (11)	0.60694 (7)	0.70543 (5)	0.0233 (2)	

### (R,R)/(S,S)-(1r,1'S,4S,E)-6'-Bromo-N-[(1r,1'S,4S)-6'-bromo-4-methoxy-4''-methyl-3'H-dispiro[cyclohexane-1,2'-indene-1',2''-imidazol-5''-yl]-4-methoxy-4''-methyl-3'H-dispiro[cyclohexane-1,2'-indene-1',2''-imidazol]-5''-imine (2) Atomic displacement parameters (Å^2^)

**Table:**

	*U*^11^	*U*^22^	*U*^33^	*U*^12^	*U*^13^	*U*^23^
Br1A	0.01661 (19)	0.0563 (3)	0.0367 (2)	−0.00006 (10)	0.00794 (11)	−0.02513 (17)
Br2	0.03585 (10)	0.02105 (9)	0.02360 (9)	0.00887 (6)	0.01305 (7)	0.00352 (6)
C1	0.0141 (6)	0.0122 (6)	0.0154 (7)	−0.0012 (5)	0.0039 (5)	0.0001 (5)
C2	0.0167 (7)	0.0152 (7)	0.0149 (7)	−0.0023 (5)	0.0034 (6)	0.0001 (5)
C3	0.0160 (7)	0.0228 (7)	0.0185 (7)	−0.0044 (6)	0.0035 (6)	−0.0001 (6)
C4	0.0179 (7)	0.0278 (8)	0.0105 (6)	−0.0081 (6)	0.0039 (5)	−0.0001 (6)
C5A	0.0174 (13)	0.0380 (12)	0.0125 (8)	−0.0138 (8)	0.0075 (8)	−0.0092 (9)
C6A	0.0165 (12)	0.0382 (11)	0.0161 (8)	−0.0104 (8)	0.0078 (8)	−0.0112 (8)
C7A	0.0149 (13)	0.0498 (12)	0.0238 (10)	−0.0120 (8)	0.0078 (9)	−0.0208 (8)
C8A	0.0242 (15)	0.0683 (16)	0.0288 (11)	−0.0214 (11)	0.0189 (11)	−0.0257 (10)
C9A	0.038 (2)	0.0692 (16)	0.0227 (10)	−0.0302 (12)	0.0220 (12)	−0.0168 (10)
C10A	0.0292 (16)	0.0499 (13)	0.0153 (8)	−0.0223 (9)	0.0109 (9)	−0.0089 (9)
C11A	0.0400 (13)	0.0461 (15)	0.0131 (13)	−0.0225 (12)	0.0099 (9)	−0.0006 (11)
C12A	0.0217 (13)	0.0293 (10)	0.0141 (8)	−0.0120 (8)	0.0051 (9)	0.0022 (7)
C13A	0.0226 (14)	0.0294 (10)	0.0242 (13)	−0.0090 (12)	−0.0001 (13)	0.0075 (9)
C14A	0.0251 (10)	0.0287 (10)	0.0314 (11)	−0.0038 (10)	0.0040 (10)	0.0105 (8)
C15A	0.0267 (11)	0.0229 (10)	0.0268 (10)	−0.0069 (9)	0.0068 (9)	0.0058 (7)
C16A	0.0220 (9)	0.0262 (10)	0.0255 (10)	−0.0090 (8)	0.0046 (8)	0.0025 (7)
C17A	0.0193 (11)	0.0241 (12)	0.0177 (8)	−0.0075 (7)	0.0040 (8)	0.0014 (8)
C18A	0.0414 (13)	0.0270 (11)	0.0520 (15)	0.0018 (9)	0.0149 (12)	0.0051 (11)
C19	0.0157 (6)	0.0112 (6)	0.0143 (6)	0.0003 (5)	0.0059 (5)	0.0009 (5)
C20	0.0154 (6)	0.0130 (6)	0.0150 (6)	0.0011 (5)	0.0061 (5)	0.0003 (5)
C21	0.0150 (7)	0.0248 (7)	0.0173 (7)	−0.0013 (6)	0.0071 (6)	−0.0019 (6)
C22	0.0142 (6)	0.0172 (7)	0.0126 (6)	−0.0025 (5)	0.0060 (5)	−0.0018 (5)
C23	0.0144 (7)	0.0199 (7)	0.0113 (6)	−0.0001 (5)	0.0051 (5)	−0.0035 (5)
C24	0.0173 (7)	0.0203 (7)	0.0149 (7)	−0.0005 (5)	0.0081 (6)	−0.0023 (5)
C25	0.0240 (7)	0.0213 (7)	0.0137 (6)	0.0044 (6)	0.0080 (6)	−0.0023 (5)
C26	0.0175 (7)	0.0289 (8)	0.0171 (7)	0.0063 (6)	0.0048 (6)	−0.0061 (6)
C27	0.0143 (7)	0.0272 (8)	0.0198 (7)	−0.0022 (6)	0.0078 (6)	−0.0086 (6)
C28	0.0171 (7)	0.0214 (7)	0.0155 (7)	−0.0033 (6)	0.0087 (6)	−0.0062 (5)
C29	0.0179 (7)	0.0208 (7)	0.0213 (7)	−0.0046 (6)	0.0115 (6)	−0.0046 (6)
C30	0.0162 (7)	0.0162 (7)	0.0147 (7)	−0.0026 (5)	0.0059 (5)	−0.0018 (5)
C31	0.0262 (8)	0.0189 (7)	0.0152 (7)	−0.0035 (6)	0.0039 (6)	−0.0006 (6)
C32	0.0318 (8)	0.0190 (7)	0.0169 (7)	−0.0045 (6)	0.0062 (6)	0.0005 (6)
C33	0.0198 (7)	0.0166 (7)	0.0207 (7)	−0.0043 (5)	0.0055 (6)	−0.0021 (6)
C34	0.0188 (7)	0.0191 (7)	0.0155 (7)	−0.0016 (6)	0.0051 (6)	−0.0026 (5)
C35	0.0171 (7)	0.0177 (7)	0.0145 (7)	−0.0002 (5)	0.0058 (6)	−0.0014 (5)
C36	0.0350 (9)	0.0188 (8)	0.0263 (8)	−0.0046 (7)	0.0038 (7)	0.0016 (6)
N1	0.0144 (6)	0.0151 (6)	0.0135 (6)	−0.0013 (4)	0.0050 (5)	−0.0004 (4)
N2	0.0139 (6)	0.0221 (6)	0.0101 (5)	−0.0041 (5)	0.0033 (5)	−0.0004 (5)
N3	0.0185 (6)	0.0228 (6)	0.0149 (6)	−0.0070 (5)	0.0034 (5)	0.0003 (5)
N4	0.0145 (5)	0.0151 (5)	0.0132 (6)	−0.0011 (4)	0.0052 (5)	−0.0010 (4)
N5	0.0142 (5)	0.0152 (6)	0.0157 (6)	0.0003 (4)	0.0071 (5)	0.0002 (4)
O1A	0.0323 (9)	0.0226 (7)	0.0399 (8)	−0.0063 (6)	0.0105 (6)	0.0047 (6)
O2	0.0238 (5)	0.0174 (5)	0.0239 (5)	−0.0050 (4)	0.0045 (4)	−0.0010 (4)

### (R,R)/(S,S)-(1r,1'S,4S,E)-6'-Bromo-N-[(1r,1'S,4S)-6'-bromo-4-methoxy-4''-methyl-3'H-dispiro[cyclohexane-1,2'-indene-1',2''-imidazol-5''-yl]-4-methoxy-4''-methyl-3'H-dispiro[cyclohexane-1,2'-indene-1',2''-imidazol]-5''-imine (2) Geometric parameters (Å, º)

**Table:**

Br1A—C7A	1.897 (2)	C16A—H16A	0.9900
Br1B—C7B	1.916 (9)	C16A—H16B	0.9900
Br2—C25	1.9043 (15)	C16A—C17A	1.529 (3)
C1—C2	1.4959 (19)	C16B—H16C	0.9900
C1—N1	1.3081 (18)	C16B—H16D	0.9900
C1—N2	1.3374 (18)	C16B—C17B	1.53 (3)
C2—C3	1.4884 (19)	C17A—H17A	0.9900
C2—N3	1.2800 (19)	C17A—H17B	0.9900
C3—H3A	0.9800	C17B—H17C	0.9900
C3—H3B	0.9800	C17B—H17D	0.9900
C3—H3C	0.9800	C18A—H18A	0.9800
C4—C5A	1.530 (3)	C18A—H18B	0.9800
C4—C5B	1.370 (18)	C18A—H18C	0.9800
C4—C12A	1.572 (3)	C18A—O1A	1.411 (3)
C4—C12B	1.597 (18)	C18B—H18D	0.9800
C4—N2	1.4509 (17)	C18B—H18E	0.9800
C4—N3	1.4759 (18)	C18B—H18F	0.9800
C5A—C6A	1.375 (3)	C18B—O1B	1.418 (19)
C5A—C10A	1.395 (2)	C19—C20	1.4940 (18)
C5B—C6B	1.3900	C19—N1	1.3853 (18)
C5B—C10B	1.3900	C19—N4	1.2983 (18)
C6B—H6B	0.9500	C20—C21	1.4833 (19)
C6B—C7B	1.3900	C20—N5	1.2837 (18)
C7B—C8B	1.3900	C21—H21A	0.9800
C8B—H8B	0.9500	C21—H21B	0.9800
C8B—C9B	1.3900	C21—H21C	0.9800
C9B—H9B	0.9500	C22—C23	1.5196 (19)
C9B—C10B	1.3900	C22—C30	1.5778 (18)
C10B—C11B	1.516 (17)	C22—N4	1.4791 (17)
C6A—H6A	0.9500	C22—N5	1.4635 (17)
C6A—C7A	1.390 (2)	C23—C24	1.386 (2)
C7A—C8A	1.384 (3)	C23—C28	1.3930 (19)
C8A—H8A	0.9500	C24—H24	0.9500
C8A—C9A	1.379 (3)	C24—C25	1.395 (2)
C9A—H9A	0.9500	C25—C26	1.385 (2)
C9A—C10A	1.397 (3)	C26—H26	0.9500
C10A—C11A	1.496 (4)	C26—C27	1.394 (2)
C11A—H11A	0.9900	C27—H27	0.9500
C11A—H11B	0.9900	C27—C28	1.389 (2)
C11A—C12A	1.557 (3)	C28—C29	1.502 (2)
C11B—H11C	0.9900	C29—H29A	0.9900
C11B—H11D	0.9900	C29—H29B	0.9900
C11B—C12B	1.558 (18)	C29—C30	1.5544 (19)
C12A—C13A	1.523 (3)	C30—C31	1.531 (2)
C12A—C17A	1.543 (3)	C30—C35	1.5427 (18)
C12B—C13B	1.531 (18)	C31—H31A	0.9900
C12B—C17B	1.560 (18)	C31—H31B	0.9900
C13A—H13A	0.9900	C31—C32	1.532 (2)
C13A—H13B	0.9900	C32—H32A	0.9900
C13A—C14A	1.532 (4)	C32—H32B	0.9900
C13B—H13C	0.9900	C32—C33	1.527 (2)
C13B—H13D	0.9900	C33—H33	1.0000
C13B—C14B	1.58 (4)	C33—C34	1.522 (2)
C14A—H14A	0.9900	C33—O2	1.4318 (17)
C14A—H14B	0.9900	C34—H34A	0.9900
C14A—C15A	1.523 (3)	C34—H34B	0.9900
C14B—H14C	0.9900	C34—C35	1.5316 (19)
C14B—H14D	0.9900	C35—H35A	0.9900
C14B—C15B	1.44 (2)	C35—H35B	0.9900
C15A—H15A	1.0000	C36—H36A	0.9800
C15A—C16A	1.510 (3)	C36—H36B	0.9800
C15A—O1A	1.427 (2)	C36—H36C	0.9800
C15B—H15B	1.0000	C36—O2	1.4232 (19)
C15B—C16B	1.49 (3)	N2—H2	0.826 (19)
C15B—O1B	1.477 (19)		
			
N1—C1—C2	125.21 (12)	C15B—C16B—H16C	109.1
N1—C1—N2	130.25 (13)	C15B—C16B—H16D	109.1
N2—C1—C2	104.43 (12)	C15B—C16B—C17B	112.3 (16)
C3—C2—C1	123.63 (12)	H16C—C16B—H16D	107.9
N3—C2—C1	111.73 (12)	C17B—C16B—H16C	109.1
N3—C2—C3	124.63 (13)	C17B—C16B—H16D	109.1
C2—C3—H3A	109.5	C12A—C17A—H17A	109.0
C2—C3—H3B	109.5	C12A—C17A—H17B	109.0
C2—C3—H3C	109.5	C16A—C17A—C12A	112.77 (17)
H3A—C3—H3B	109.5	C16A—C17A—H17A	109.0
H3A—C3—H3C	109.5	C16A—C17A—H17B	109.0
H3B—C3—H3C	109.5	H17A—C17A—H17B	107.8
C5A—C4—C12A	101.9 (2)	C12B—C17B—H17C	108.1
C5B—C4—C12B	109.6 (18)	C12B—C17B—H17D	108.1
C5B—C4—N2	115.9 (15)	C16B—C17B—C12B	116.9 (16)
C5B—C4—N3	109 (2)	C16B—C17B—H17C	108.1
N2—C4—C5A	115.42 (19)	C16B—C17B—H17D	108.1
N2—C4—C12A	114.73 (16)	H17C—C17B—H17D	107.3
N2—C4—C12B	112.3 (10)	H18A—C18A—H18B	109.5
N2—C4—N3	104.06 (11)	H18A—C18A—H18C	109.5
N3—C4—C5A	109.3 (2)	H18B—C18A—H18C	109.5
N3—C4—C12A	111.57 (14)	O1A—C18A—H18A	109.5
N3—C4—C12B	105.2 (9)	O1A—C18A—H18B	109.5
C6A—C5A—C4	127.7 (2)	O1A—C18A—H18C	109.5
C6A—C5A—C10A	123.04 (17)	H18D—C18B—H18E	109.5
C10A—C5A—C4	109.2 (2)	H18D—C18B—H18F	109.5
C4—C5B—C6B	129.4 (15)	H18E—C18B—H18F	109.5
C4—C5B—C10B	110.6 (15)	O1B—C18B—H18D	109.5
C6B—C5B—C10B	120.0	O1B—C18B—H18E	109.5
C5B—C6B—H6B	120.0	O1B—C18B—H18F	109.5
C5B—C6B—C7B	120.0	N1—C19—C20	121.30 (12)
C7B—C6B—H6B	120.0	N4—C19—C20	110.01 (12)
C6B—C7B—Br1B	119.7 (7)	N4—C19—N1	128.65 (12)
C6B—C7B—C8B	120.0	C21—C20—C19	124.80 (12)
C8B—C7B—Br1B	120.3 (7)	N5—C20—C19	110.24 (12)
C7B—C8B—H8B	120.0	N5—C20—C21	124.96 (12)
C9B—C8B—C7B	120.0	C20—C21—H21A	109.5
C9B—C8B—H8B	120.0	C20—C21—H21B	109.5
C8B—C9B—H9B	120.0	C20—C21—H21C	109.5
C8B—C9B—C10B	120.0	H21A—C21—H21B	109.5
C10B—C9B—H9B	120.0	H21A—C21—H21C	109.5
C5B—C10B—C11B	109.3 (18)	H21B—C21—H21C	109.5
C9B—C10B—C5B	120.0	C23—C22—C30	102.40 (11)
C9B—C10B—C11B	130.5 (19)	N4—C22—C23	109.30 (11)
C5A—C6A—H6A	121.8	N4—C22—C30	110.81 (10)
C5A—C6A—C7A	116.49 (17)	N5—C22—C23	112.87 (11)
C7A—C6A—H6A	121.8	N5—C22—C30	113.26 (11)
C6A—C7A—Br1A	118.23 (17)	N5—C22—N4	108.11 (10)
C8A—C7A—Br1A	119.18 (18)	C24—C23—C22	127.95 (12)
C8A—C7A—C6A	122.6 (2)	C24—C23—C28	122.05 (13)
C7A—C8A—H8A	120.2	C28—C23—C22	109.99 (12)
C9A—C8A—C7A	119.57 (18)	C23—C24—H24	121.5
C9A—C8A—H8A	120.2	C23—C24—C25	117.00 (13)
C8A—C9A—H9A	120.1	C25—C24—H24	121.5
C8A—C9A—C10A	119.84 (19)	C24—C25—Br2	118.46 (11)
C10A—C9A—H9A	120.1	C26—C25—Br2	119.30 (11)
C5A—C10A—C9A	118.5 (2)	C26—C25—C24	122.24 (14)
C5A—C10A—C11A	110.7 (3)	C25—C26—H26	120.2
C9A—C10A—C11A	130.8 (3)	C25—C26—C27	119.60 (13)
C10A—C11A—H11A	111.1	C27—C26—H26	120.2
C10A—C11A—H11B	111.1	C26—C27—H27	120.3
C10A—C11A—C12A	103.2 (3)	C28—C27—C26	119.34 (13)
H11A—C11A—H11B	109.1	C28—C27—H27	120.3
C12A—C11A—H11A	111.1	C23—C28—C29	110.39 (12)
C12A—C11A—H11B	111.1	C27—C28—C23	119.76 (14)
C10B—C11B—H11C	110.5	C27—C28—C29	129.84 (13)
C10B—C11B—H11D	110.5	C28—C29—H29A	111.0
C10B—C11B—C12B	106.1 (18)	C28—C29—H29B	111.0
H11C—C11B—H11D	108.7	C28—C29—C30	103.86 (11)
C12B—C11B—H11C	110.5	H29A—C29—H29B	109.0
C12B—C11B—H11D	110.5	C30—C29—H29A	111.0
C11A—C12A—C4	101.9 (2)	C30—C29—H29B	111.0
C13A—C12A—C4	111.3 (2)	C29—C30—C22	102.12 (11)
C13A—C12A—C11A	115.1 (3)	C31—C30—C22	112.43 (11)
C13A—C12A—C17A	110.0 (2)	C31—C30—C29	113.56 (12)
C17A—C12A—C4	108.8 (2)	C31—C30—C35	107.73 (11)
C17A—C12A—C11A	109.5 (3)	C35—C30—C22	110.21 (11)
C11B—C12B—C4	98.0 (15)	C35—C30—C29	110.76 (11)
C11B—C12B—C17B	104 (3)	C30—C31—H31A	109.3
C13B—C12B—C4	117 (2)	C30—C31—H31B	109.3
C13B—C12B—C11B	115 (2)	C30—C31—C32	111.46 (12)
C13B—C12B—C17B	107.8 (18)	H31A—C31—H31B	108.0
C17B—C12B—C4	114.4 (18)	C32—C31—H31A	109.3
C12A—C13A—H13A	109.1	C32—C31—H31B	109.3
C12A—C13A—H13B	109.1	C31—C32—H32A	109.2
C12A—C13A—C14A	112.4 (2)	C31—C32—H32B	109.2
H13A—C13A—H13B	107.9	H32A—C32—H32B	107.9
C14A—C13A—H13A	109.1	C33—C32—C31	111.93 (12)
C14A—C13A—H13B	109.1	C33—C32—H32A	109.2
C12B—C13B—H13C	108.0	C33—C32—H32B	109.2
C12B—C13B—H13D	108.0	C32—C33—H33	109.1
C12B—C13B—C14B	117 (2)	C34—C33—C32	111.51 (12)
H13C—C13B—H13D	107.2	C34—C33—H33	109.1
C14B—C13B—H13C	108.0	O2—C33—C32	110.57 (12)
C14B—C13B—H13D	108.0	O2—C33—H33	109.1
C13A—C14A—H14A	109.5	O2—C33—C34	107.43 (11)
C13A—C14A—H14B	109.5	C33—C34—H34A	109.2
H14A—C14A—H14B	108.1	C33—C34—H34B	109.2
C15A—C14A—C13A	110.71 (17)	C33—C34—C35	112.26 (11)
C15A—C14A—H14A	109.5	H34A—C34—H34B	107.9
C15A—C14A—H14B	109.5	C35—C34—H34A	109.2
C13B—C14B—H14C	109.2	C35—C34—H34B	109.2
C13B—C14B—H14D	109.2	C30—C35—H35A	109.1
H14C—C14B—H14D	107.9	C30—C35—H35B	109.1
C15B—C14B—C13B	112.1 (18)	C34—C35—C30	112.46 (11)
C15B—C14B—H14C	109.2	C34—C35—H35A	109.1
C15B—C14B—H14D	109.2	C34—C35—H35B	109.1
C14A—C15A—H15A	109.0	H35A—C35—H35B	107.8
C16A—C15A—C14A	110.03 (18)	H36A—C36—H36B	109.5
C16A—C15A—H15A	109.0	H36A—C36—H36C	109.5
O1A—C15A—C14A	113.11 (17)	H36B—C36—H36C	109.5
O1A—C15A—H15A	109.0	O2—C36—H36A	109.5
O1A—C15A—C16A	106.73 (18)	O2—C36—H36B	109.5
C14B—C15B—H15B	108.8	O2—C36—H36C	109.5
C14B—C15B—C16B	111.8 (14)	C1—N1—C19	116.17 (12)
C14B—C15B—O1B	108.8 (14)	C1—N2—C4	111.11 (12)
C16B—C15B—H15B	108.8	C1—N2—H2	119.9 (12)
O1B—C15B—H15B	108.8	C4—N2—H2	128.8 (12)
O1B—C15B—C16B	109.8 (13)	C2—N3—C4	108.41 (12)
C15A—C16A—H16A	109.3	C19—N4—C22	105.32 (11)
C15A—C16A—H16B	109.3	C20—N5—C22	106.28 (11)
C15A—C16A—C17A	111.51 (16)	C18A—O1A—C15A	114.26 (18)
H16A—C16A—H16B	108.0	C18B—O1B—C15B	115.5 (11)
C17A—C16A—H16A	109.3	C36—O2—C33	113.31 (11)
C17A—C16A—H16B	109.3		
			
Br1A—C7A—C8A—C9A	179.2 (4)	C22—C23—C24—C25	178.69 (13)
Br1B—C7B—C8B—C9B	−178 (3)	C22—C23—C28—C27	−178.89 (12)
Br2—C25—C26—C27	178.87 (10)	C22—C23—C28—C29	1.91 (15)
C1—C2—N3—C4	1.44 (16)	C22—C30—C31—C32	−179.77 (12)
C2—C1—N1—C19	−170.60 (12)	C22—C30—C35—C34	179.62 (11)
C2—C1—N2—C4	5.17 (15)	C23—C22—C30—C29	31.83 (12)
C3—C2—N3—C4	−179.96 (13)	C23—C22—C30—C31	153.90 (11)
C4—C5A—C6A—C7A	−179.2 (5)	C23—C22—C30—C35	−85.90 (12)
C4—C5A—C10A—C9A	−180.0 (4)	C23—C22—N4—C19	125.11 (12)
C4—C5A—C10A—C11A	1.7 (5)	C23—C22—N5—C20	−122.43 (12)
C4—C5B—C6B—C7B	178 (5)	C23—C24—C25—Br2	−179.34 (10)
C4—C5B—C10B—C9B	−179 (4)	C23—C24—C25—C26	0.1 (2)
C4—C5B—C10B—C11B	7 (4)	C23—C28—C29—C30	19.18 (14)
C4—C12A—C13A—C14A	173.7 (2)	C24—C23—C28—C27	−0.3 (2)
C4—C12A—C17A—C16A	−173.89 (18)	C24—C23—C28—C29	−179.47 (12)
C4—C12B—C13B—C14B	172 (2)	C24—C25—C26—C27	−0.5 (2)
C4—C12B—C17B—C16B	−174.2 (17)	C25—C26—C27—C28	0.6 (2)
C5A—C4—C12A—C11A	34.2 (4)	C26—C27—C28—C23	−0.2 (2)
C5A—C4—C12A—C13A	157.4 (3)	C26—C27—C28—C29	178.82 (13)
C5A—C4—C12A—C17A	−81.3 (3)	C27—C28—C29—C30	−159.92 (14)
C5A—C4—N2—C1	−124.3 (3)	C28—C23—C24—C25	0.3 (2)
C5A—C4—N3—C2	125.48 (18)	C28—C29—C30—C22	−31.12 (13)
C5A—C6A—C7A—Br1A	−179.6 (4)	C28—C29—C30—C31	−152.41 (11)
C5A—C6A—C7A—C8A	−1.2 (2)	C28—C29—C30—C35	86.22 (13)
C5A—C10A—C11A—C12A	20.8 (5)	C29—C30—C31—C32	−64.44 (16)
C5B—C4—C12B—C11B	25 (3)	C29—C30—C35—C34	67.36 (15)
C5B—C4—C12B—C13B	149 (3)	C30—C22—C23—C24	159.75 (13)
C5B—C4—C12B—C17B	−84 (3)	C30—C22—C23—C28	−21.74 (14)
C5B—C4—N2—C1	−124 (2)	C30—C22—N4—C19	−122.77 (12)
C5B—C4—N3—C2	125.9 (11)	C30—C22—N5—C20	121.78 (12)
C5B—C6B—C7B—Br1B	178 (3)	C30—C31—C32—C33	−57.70 (17)
C5B—C6B—C7B—C8B	0.0	C31—C30—C35—C34	−57.38 (15)
C5B—C10B—C11B—C12B	10 (4)	C31—C32—C33—C34	52.46 (17)
C6B—C5B—C10B—C9B	0.0	C31—C32—C33—O2	171.92 (12)
C6B—C5B—C10B—C11B	−175 (4)	C32—C33—C34—C35	−50.88 (16)
C6B—C7B—C8B—C9B	0.0	C32—C33—O2—C36	81.23 (16)
C7B—C8B—C9B—C10B	0.0	C33—C34—C35—C30	54.70 (16)
C8B—C9B—C10B—C5B	0.0	C34—C33—O2—C36	−156.88 (12)
C8B—C9B—C10B—C11B	174 (5)	C35—C30—C31—C32	58.61 (16)
C9B—C10B—C11B—C12B	−164 (3)	N1—C1—C2—C3	−6.3 (2)
C10B—C5B—C6B—C7B	0.0	N1—C1—C2—N3	172.30 (13)
C10B—C11B—C12B—C4	−20 (4)	N1—C1—N2—C4	−171.09 (14)
C10B—C11B—C12B—C13B	−145 (3)	N1—C19—C20—C21	2.0 (2)
C10B—C11B—C12B—C17B	98 (4)	N1—C19—C20—N5	−176.99 (12)
C6A—C5A—C10A—C9A	0.1 (3)	N1—C19—N4—C22	175.98 (13)
C6A—C5A—C10A—C11A	−178.3 (5)	N2—C1—C2—C3	177.18 (13)
C6A—C7A—C8A—C9A	0.7 (3)	N2—C1—C2—N3	−4.21 (16)
C7A—C8A—C9A—C10A	0.2 (3)	N2—C1—N1—C19	5.0 (2)
C8A—C9A—C10A—C5A	−0.6 (3)	N2—C4—C5A—C6A	31.9 (4)
C8A—C9A—C10A—C11A	177.4 (6)	N2—C4—C5A—C10A	−148.1 (2)
C9A—C10A—C11A—C12A	−157.3 (4)	N2—C4—C5B—C6B	32 (4)
C10A—C5A—C6A—C7A	0.8 (2)	N2—C4—C5B—C10B	−149.3 (17)
C10A—C11A—C12A—C4	−33.7 (5)	N2—C4—C12A—C11A	159.7 (3)
C10A—C11A—C12A—C13A	−154.2 (4)	N2—C4—C12A—C13A	−77.2 (3)
C10A—C11A—C12A—C17A	81.4 (4)	N2—C4—C12A—C17A	44.1 (2)
C11A—C12A—C13A—C14A	−71.1 (4)	N2—C4—C12B—C11B	156 (2)
C11A—C12A—C17A—C16A	75.6 (2)	N2—C4—C12B—C13B	−81 (2)
C11B—C12B—C13B—C14B	−74 (3)	N2—C4—C12B—C17B	46 (2)
C11B—C12B—C17B—C16B	80 (2)	N2—C4—N3—C2	1.64 (16)
C12A—C4—C5A—C6A	156.9 (3)	N3—C4—C5A—C6A	−85.0 (4)
C12A—C4—C5A—C10A	−23.1 (3)	N3—C4—C5A—C10A	95.0 (3)
C12A—C4—N2—C1	117.70 (15)	N3—C4—C5B—C6B	−85 (3)
C12A—C4—N3—C2	−122.61 (16)	N3—C4—C5B—C10B	94 (2)
C12A—C13A—C14A—C15A	−57.2 (3)	N3—C4—C12A—C11A	−82.3 (3)
C12B—C4—C5B—C6B	161 (3)	N3—C4—C12A—C13A	40.8 (3)
C12B—C4—C5B—C10B	−21 (3)	N3—C4—C12A—C17A	162.13 (15)
C12B—C4—N2—C1	108.8 (10)	N3—C4—C12B—C11B	−92 (2)
C12B—C4—N3—C2	−116.6 (11)	N3—C4—C12B—C13B	31 (2)
C12B—C13B—C14B—C15B	−51 (3)	N3—C4—C12B—C17B	159.0 (16)
C13A—C12A—C17A—C16A	−51.7 (3)	N3—C4—N2—C1	−4.47 (16)
C13A—C14A—C15A—C16A	58.1 (3)	N4—C19—C20—C21	179.78 (13)
C13A—C14A—C15A—O1A	177.4 (2)	N4—C19—C20—N5	0.82 (16)
C13B—C12B—C17B—C16B	−42 (3)	N4—C19—N1—C1	−10.6 (2)
C13B—C14B—C15B—C16B	55 (2)	N4—C22—C23—C24	−82.71 (16)
C13B—C14B—C15B—O1B	176.8 (17)	N4—C22—C23—C28	95.80 (13)
C14A—C15A—C16A—C17A	−57.1 (2)	N4—C22—C30—C29	−84.63 (12)
C14A—C15A—O1A—C18A	74.0 (3)	N4—C22—C30—C31	37.44 (15)
C14B—C15B—C16B—C17B	−56 (2)	N4—C22—C30—C35	157.64 (11)
C14B—C15B—O1B—C18B	161.4 (13)	N4—C22—N5—C20	−1.40 (14)
C15A—C16A—C17A—C12A	54.7 (2)	N5—C22—C23—C24	37.64 (19)
C15B—C16B—C17B—C12B	51 (2)	N5—C22—C23—C28	−143.85 (12)
C16A—C15A—O1A—C18A	−164.90 (18)	N5—C22—C30—C29	153.68 (11)
C16B—C15B—O1B—C18B	−75.9 (16)	N5—C22—C30—C31	−84.25 (14)
C17A—C12A—C13A—C14A	53.0 (3)	N5—C22—C30—C35	35.95 (15)
C17B—C12B—C13B—C14B	41 (3)	N5—C22—N4—C19	1.89 (14)
C19—C20—N5—C22	0.43 (14)	O1A—C15A—C16A—C17A	179.84 (15)
C20—C19—N1—C1	166.76 (12)	O1B—C15B—C16B—C17B	−177.2 (14)
C20—C19—N4—C22	−1.62 (14)	O2—C33—C34—C35	−172.18 (11)
C21—C20—N5—C22	−178.53 (13)		

### (R,R)/(S,S)-(1r,1'S,4S,E)-6'-Bromo-N-[(1r,1'S,4S)-6'-bromo-4-methoxy-4''-methyl-3'H-dispiro[cyclohexane-1,2'-indene-1',2''-imidazol-5''-yl]-4-methoxy-4''-methyl-3'H-dispiro[cyclohexane-1,2'-indene-1',2''-imidazol]-5''-imine (2) Hydrogen-bond geometry (Å, º)

**Table:**

*D*—H···*A*	*D*—H	H···*A*	*D*···*A*	*D*—H···*A*
N2—H2···N4	0.826 (19)	2.064 (18)	2.6456 (16)	127.2 (15)

### (R,S)/(S,R)-(1r,1'S,4S,E)-6'-Bromo-N-[(1r,1'S,4S)-6'-bromo-4-methoxy-4''-methyl-3'H-dispiro[cyclohexane-1,2'-indene-1',2''-imidazol-5''-yl]-4-methoxy-4''-methyl-3'H-dispiro[cyclohexane-1,2'-indene-1',2''-imidazol]-5''-imine (1) Crystal data

**Table:**

C_36_H_41_Br_2_N_5_O_2_	*F*(000) = 1512
*M**_r_* = 735.56	*D*_x_ = 1.424 Mg m^−^^3^
Monoclinic, *P*2_1_/*n*	Cu *K*α radiation, λ = 1.54178 Å
*a* = 13.19297 (6) Å	Cell parameters from 74158 reflections
*b* = 17.60010 (8) Å	θ = 2.5–70.4°
*c* = 15.25349 (8) Å	µ = 3.30 mm^−^^1^
β = 104.4018 (5)°	*T* = 100 K
*V* = 3430.52 (3) Å^3^	Block, colourless
*Z* = 4	0.31 × 0.07 × 0.05 mm

### (R,S)/(S,R)-(1r,1'S,4S,E)-6'-Bromo-N-[(1r,1'S,4S)-6'-bromo-4-methoxy-4''-methyl-3'H-dispiro[cyclohexane-1,2'-indene-1',2''-imidazol-5''-yl]-4-methoxy-4''-methyl-3'H-dispiro[cyclohexane-1,2'-indene-1',2''-imidazol]-5''-imine (1) Data collection

**Table:**

Rigaku 007HF equipped with Varimax confocal mirrors and an AFC11 goniometer and HyPix 6000 detector diffractometer	6285 independent reflections
Radiation source: Rotating anode, Rigaku 007 HF	6257 reflections with *I* > 2σ(*I*)
Mirror monochromator	*R*_int_ = 0.023
Detector resolution: 10 pixels mm^-1^	θ_max_ = 68.3°, θ_min_ = 3.9°
profile data from ω–scans	*h* = −15→15
Absorption correction: gaussian (CrysAlisPro; Rigaku OD, 2019)	*k* = −21→21
*T*_min_ = 0.654, *T*_max_ = 1.000	*l* = −18→18
97773 measured reflections	

### (R,S)/(S,R)-(1r,1'S,4S,E)-6'-Bromo-N-[(1r,1'S,4S)-6'-bromo-4-methoxy-4''-methyl-3'H-dispiro[cyclohexane-1,2'-indene-1',2''-imidazol-5''-yl]-4-methoxy-4''-methyl-3'H-dispiro[cyclohexane-1,2'-indene-1',2''-imidazol]-5''-imine (1) Refinement

**Table:**

Refinement on *F*^2^	Primary atom site location: dual
Least-squares matrix: full	Hydrogen site location: mixed
*R*[*F*^2^ > 2σ(*F*^2^)] = 0.024	H atoms treated by a mixture of independent and constrained refinement
*wR*(*F*^2^) = 0.057	*w* = 1/[σ^2^(*F*_o_^2^) + (0.0248*P*)^2^ + 2.9185*P*] where *P* = (*F*_o_^2^ + 2*F*_c_^2^)/3
*S* = 1.06	(Δ/σ)_max_ = 0.001
6285 reflections	Δρ_max_ = 0.33 e Å^−^^3^
413 parameters	Δρ_min_ = −0.44 e Å^−^^3^
1 restraint	

### (R,S)/(S,R)-(1r,1'S,4S,E)-6'-Bromo-N-[(1r,1'S,4S)-6'-bromo-4-methoxy-4''-methyl-3'H-dispiro[cyclohexane-1,2'-indene-1',2''-imidazol-5''-yl]-4-methoxy-4''-methyl-3'H-dispiro[cyclohexane-1,2'-indene-1',2''-imidazol]-5''-imine (1) Special details

**Table:**

Geometry. All esds (except the esd in the dihedral angle between two l.s. planes) are estimated using the full covariance matrix. The cell esds are taken into account individually in the estimation of esds in distances, angles and torsion angles; correlations between esds in cell parameters are only used when they are defined by crystal symmetry. An approximate (isotropic) treatment of cell esds is used for estimating esds involving l.s. planes.
Refinement. This diastereoisomer has crystallised in the centrosymmetric space group P2_1_/n; meaning that both SS and RR forms of the API must be present in equal amounts within the crystal.

### (R,S)/(S,R)-(1r,1'S,4S,E)-6'-Bromo-N-[(1r,1'S,4S)-6'-bromo-4-methoxy-4''-methyl-3'H-dispiro[cyclohexane-1,2'-indene-1',2''-imidazol-5''-yl]-4-methoxy-4''-methyl-3'H-dispiro[cyclohexane-1,2'-indene-1',2''-imidazol]-5''-imine (1) Fractional atomic coordinates and isotropic or equivalent isotropic displacement parameters (Å^2^)

**Table:**

	*x*	*y*	*z*	*U*_iso_*/*U*_eq_	
Br1	0.57538 (2)	0.34738 (2)	0.89948 (2)	0.02177 (5)	
Br2	0.14814 (2)	0.66375 (2)	0.24065 (2)	0.02990 (6)	
C1	0.15668 (11)	0.50387 (8)	0.69473 (9)	0.0129 (3)	
C2	0.13914 (11)	0.52942 (8)	0.78320 (10)	0.0144 (3)	
C3	0.04172 (12)	0.51294 (10)	0.81180 (11)	0.0214 (3)	
H3A	0.048466	0.532334	0.873213	0.032*	
H3B	0.030082	0.457923	0.810962	0.032*	
H3C	−0.017676	0.537693	0.770109	0.032*	
C4	0.29513 (11)	0.57251 (8)	0.77417 (9)	0.0133 (3)	
C5	0.39917 (11)	0.54110 (8)	0.82716 (9)	0.0140 (3)	
C6	0.43135 (12)	0.46632 (9)	0.83361 (10)	0.0162 (3)	
H6	0.388386	0.427279	0.800815	0.019*	
C7	0.52897 (12)	0.45036 (9)	0.88989 (10)	0.0173 (3)	
C8	0.59218 (12)	0.50677 (9)	0.93894 (10)	0.0185 (3)	
H8	0.657774	0.494104	0.978454	0.022*	
C9	0.55861 (12)	0.58207 (9)	0.92970 (10)	0.0176 (3)	
H9	0.601620	0.621205	0.962229	0.021*	
C10	0.46163 (12)	0.59959 (9)	0.87251 (10)	0.0157 (3)	
C11	0.40713 (12)	0.67508 (9)	0.84824 (10)	0.0175 (3)	
H11A	0.456675	0.714295	0.837849	0.021*	
H11B	0.374767	0.692667	0.896646	0.021*	
C12	0.32230 (11)	0.65767 (8)	0.75962 (10)	0.0136 (3)	
C13	0.22456 (11)	0.70763 (8)	0.74388 (11)	0.0174 (3)	
H13A	0.168199	0.684004	0.696541	0.021*	
H13B	0.200517	0.709635	0.800360	0.021*	
C14	0.24256 (13)	0.78862 (9)	0.71501 (13)	0.0251 (4)	
H14A	0.175330	0.816570	0.701148	0.030*	
H14B	0.291377	0.814993	0.765677	0.030*	
C15	0.28730 (12)	0.78963 (9)	0.63259 (12)	0.0217 (3)	
H15	0.236081	0.765257	0.580750	0.026*	
C16	0.38937 (12)	0.74506 (8)	0.65217 (11)	0.0177 (3)	
H16A	0.441114	0.769143	0.702812	0.021*	
H16B	0.418255	0.745545	0.598164	0.021*	
C17	0.36986 (11)	0.66312 (8)	0.67692 (10)	0.0145 (3)	
H17A	0.436898	0.634951	0.690158	0.017*	
H17B	0.321797	0.638505	0.624337	0.017*	
C18	0.33503 (15)	0.87778 (11)	0.53124 (14)	0.0355 (4)	
H18A	0.294890	0.844671	0.483397	0.053*	
H18B	0.409455	0.864737	0.543539	0.053*	
H18C	0.325097	0.930878	0.511642	0.053*	
C19	0.12763 (11)	0.43884 (8)	0.56219 (9)	0.0124 (3)	
C20	0.07361 (11)	0.38220 (8)	0.49353 (9)	0.0123 (3)	
C21	−0.02914 (11)	0.34673 (8)	0.49334 (10)	0.0159 (3)	
H21A	−0.085343	0.383654	0.471467	0.024*	
H21B	−0.029155	0.330987	0.554960	0.024*	
H21C	−0.040429	0.302236	0.453450	0.024*	
C22	0.22276 (11)	0.41636 (8)	0.46271 (9)	0.0117 (3)	
C23	0.23216 (11)	0.46484 (8)	0.38289 (9)	0.0123 (3)	
C24	0.18603 (11)	0.53442 (8)	0.35623 (10)	0.0158 (3)	
H24	0.141328	0.558250	0.388096	0.019*	
C25	0.20823 (11)	0.56779 (9)	0.28060 (11)	0.0189 (3)	
C26	0.27239 (12)	0.53353 (10)	0.23273 (11)	0.0212 (3)	
H26	0.285223	0.557722	0.180869	0.025*	
C27	0.31778 (12)	0.46373 (9)	0.26092 (10)	0.0196 (3)	
H27	0.362047	0.439791	0.228716	0.023*	
C28	0.29774 (11)	0.42925 (8)	0.33682 (9)	0.0138 (3)	
C29	0.33843 (11)	0.35584 (8)	0.38287 (10)	0.0144 (3)	
H29A	0.412775	0.347934	0.383041	0.017*	
H29B	0.296824	0.312047	0.352846	0.017*	
C30	0.32551 (11)	0.36684 (8)	0.48025 (9)	0.0118 (3)	
C31	0.31641 (12)	0.29247 (8)	0.52930 (10)	0.0166 (3)	
H31A	0.260115	0.261062	0.491214	0.020*	
H31B	0.296945	0.303725	0.586573	0.020*	
C32	0.41931 (12)	0.24756 (9)	0.55019 (11)	0.0196 (3)	
H32A	0.411332	0.201096	0.584435	0.023*	
H32B	0.435041	0.231742	0.492700	0.023*	
C33	0.51027 (12)	0.29466 (9)	0.60523 (11)	0.0219 (3)	
H33	0.497643	0.305715	0.665943	0.026*	
C34	0.52114 (12)	0.36922 (9)	0.55827 (11)	0.0189 (3)	
H34A	0.541834	0.358860	0.501331	0.023*	
H34B	0.576778	0.400299	0.597697	0.023*	
C35	0.41793 (11)	0.41339 (8)	0.53713 (10)	0.0152 (3)	
H35A	0.426159	0.460335	0.503813	0.018*	
H35B	0.401738	0.428523	0.594668	0.018*	
C36	0.62272 (17)	0.19682 (12)	0.68103 (15)	0.0448 (5)	
H36A	0.573730	0.155573	0.657157	0.067*	
H36B	0.609719	0.215570	0.737741	0.067*	
H36C	0.694655	0.177843	0.692376	0.067*	
N1	0.09138 (9)	0.46044 (7)	0.63455 (8)	0.0131 (2)	
N2	0.24915 (9)	0.53150 (7)	0.69163 (8)	0.0122 (2)	
H2	0.2733 (13)	0.5217 (10)	0.6481 (11)	0.015*	
N3	0.21762 (9)	0.56719 (7)	0.82906 (8)	0.0151 (3)	
N4	0.21425 (9)	0.45973 (7)	0.54149 (8)	0.0124 (2)	
N5	0.12793 (9)	0.36819 (7)	0.43652 (8)	0.0130 (2)	
O1	0.29971 (10)	0.86771 (7)	0.61123 (9)	0.0308 (3)	
O2	0.60841 (9)	0.25660 (7)	0.61737 (8)	0.0303 (3)	

### (R,S)/(S,R)-(1r,1'S,4S,E)-6'-Bromo-N-[(1r,1'S,4S)-6'-bromo-4-methoxy-4''-methyl-3'H-dispiro[cyclohexane-1,2'-indene-1',2''-imidazol-5''-yl]-4-methoxy-4''-methyl-3'H-dispiro[cyclohexane-1,2'-indene-1',2''-imidazol]-5''-imine (1) Atomic displacement parameters (Å^2^)

**Table:**

	*U*^11^	*U*^22^	*U*^33^	*U*^12^	*U*^13^	*U*^23^
Br1	0.02290 (9)	0.01996 (9)	0.02374 (9)	0.00478 (6)	0.00824 (7)	0.00669 (6)
Br2	0.02056 (9)	0.02260 (10)	0.04550 (12)	0.00515 (7)	0.00627 (8)	0.01708 (8)
C1	0.0132 (7)	0.0124 (7)	0.0139 (7)	0.0012 (5)	0.0048 (5)	−0.0003 (5)
C2	0.0155 (7)	0.0142 (7)	0.0147 (7)	0.0000 (5)	0.0063 (6)	−0.0019 (5)
C3	0.0190 (8)	0.0274 (8)	0.0211 (8)	−0.0053 (6)	0.0113 (6)	−0.0069 (7)
C4	0.0136 (7)	0.0146 (7)	0.0124 (7)	−0.0023 (5)	0.0047 (5)	−0.0039 (5)
C5	0.0144 (7)	0.0169 (7)	0.0117 (6)	−0.0030 (6)	0.0051 (5)	−0.0012 (5)
C6	0.0175 (7)	0.0166 (7)	0.0154 (7)	−0.0039 (6)	0.0056 (6)	−0.0005 (6)
C7	0.0187 (7)	0.0196 (8)	0.0159 (7)	0.0004 (6)	0.0086 (6)	0.0035 (6)
C8	0.0137 (7)	0.0288 (8)	0.0136 (7)	−0.0005 (6)	0.0046 (6)	0.0023 (6)
C9	0.0163 (7)	0.0232 (8)	0.0137 (7)	−0.0057 (6)	0.0045 (6)	−0.0030 (6)
C10	0.0167 (7)	0.0184 (7)	0.0130 (7)	−0.0037 (6)	0.0056 (6)	−0.0025 (6)
C11	0.0183 (7)	0.0163 (7)	0.0168 (7)	−0.0038 (6)	0.0026 (6)	−0.0059 (6)
C12	0.0139 (7)	0.0121 (7)	0.0153 (7)	−0.0013 (5)	0.0044 (6)	−0.0039 (5)
C13	0.0152 (7)	0.0157 (7)	0.0229 (7)	0.0009 (6)	0.0078 (6)	−0.0045 (6)
C14	0.0223 (8)	0.0148 (8)	0.0410 (10)	0.0037 (6)	0.0129 (7)	−0.0023 (7)
C15	0.0190 (8)	0.0123 (7)	0.0334 (9)	0.0004 (6)	0.0057 (7)	0.0040 (6)
C16	0.0165 (7)	0.0137 (7)	0.0237 (8)	−0.0008 (6)	0.0068 (6)	0.0007 (6)
C17	0.0144 (7)	0.0132 (7)	0.0171 (7)	0.0007 (5)	0.0062 (6)	−0.0012 (5)
C18	0.0284 (9)	0.0273 (10)	0.0515 (12)	0.0043 (8)	0.0111 (8)	0.0194 (9)
C19	0.0114 (7)	0.0118 (7)	0.0135 (7)	−0.0003 (5)	0.0020 (5)	0.0002 (5)
C20	0.0121 (7)	0.0122 (7)	0.0122 (6)	−0.0002 (5)	0.0021 (5)	0.0004 (5)
C21	0.0124 (7)	0.0169 (7)	0.0183 (7)	−0.0033 (6)	0.0040 (6)	−0.0031 (6)
C22	0.0119 (7)	0.0129 (7)	0.0106 (6)	−0.0026 (5)	0.0032 (5)	−0.0029 (5)
C23	0.0100 (6)	0.0149 (7)	0.0114 (6)	−0.0030 (5)	0.0014 (5)	−0.0005 (5)
C24	0.0109 (7)	0.0174 (7)	0.0190 (7)	−0.0005 (6)	0.0033 (6)	−0.0003 (6)
C25	0.0113 (7)	0.0187 (8)	0.0245 (8)	−0.0005 (6)	0.0005 (6)	0.0061 (6)
C26	0.0171 (7)	0.0278 (9)	0.0193 (7)	−0.0013 (6)	0.0055 (6)	0.0090 (6)
C27	0.0177 (7)	0.0271 (8)	0.0155 (7)	0.0024 (6)	0.0072 (6)	0.0024 (6)
C28	0.0128 (7)	0.0165 (7)	0.0117 (6)	−0.0011 (5)	0.0025 (5)	−0.0006 (5)
C29	0.0158 (7)	0.0157 (7)	0.0127 (7)	0.0015 (6)	0.0050 (5)	−0.0011 (5)
C30	0.0120 (7)	0.0122 (7)	0.0114 (6)	−0.0005 (5)	0.0033 (5)	−0.0004 (5)
C31	0.0183 (7)	0.0153 (7)	0.0171 (7)	−0.0015 (6)	0.0059 (6)	0.0021 (6)
C32	0.0223 (8)	0.0158 (7)	0.0204 (7)	0.0020 (6)	0.0050 (6)	0.0051 (6)
C33	0.0211 (8)	0.0235 (8)	0.0184 (7)	0.0055 (6)	−0.0002 (6)	0.0026 (6)
C34	0.0137 (7)	0.0205 (8)	0.0202 (7)	−0.0003 (6)	−0.0001 (6)	−0.0012 (6)
C35	0.0138 (7)	0.0153 (7)	0.0154 (7)	−0.0013 (6)	0.0017 (5)	−0.0018 (6)
C36	0.0391 (11)	0.0338 (11)	0.0487 (12)	0.0068 (9)	−0.0132 (9)	0.0160 (9)
N1	0.0121 (6)	0.0147 (6)	0.0133 (6)	−0.0017 (5)	0.0047 (5)	−0.0032 (5)
N2	0.0123 (6)	0.0135 (6)	0.0119 (6)	−0.0016 (5)	0.0050 (5)	−0.0042 (5)
N3	0.0166 (6)	0.0160 (6)	0.0144 (6)	−0.0010 (5)	0.0068 (5)	−0.0025 (5)
N4	0.0129 (6)	0.0133 (6)	0.0119 (6)	−0.0009 (5)	0.0046 (5)	−0.0021 (5)
N5	0.0112 (6)	0.0140 (6)	0.0130 (6)	−0.0023 (5)	0.0016 (5)	−0.0003 (5)
O1	0.0299 (6)	0.0148 (6)	0.0502 (8)	0.0034 (5)	0.0149 (6)	0.0090 (5)
O2	0.0235 (6)	0.0282 (7)	0.0333 (7)	0.0095 (5)	−0.0042 (5)	0.0053 (5)

### (R,S)/(S,R)-(1r,1'S,4S,E)-6'-Bromo-N-[(1r,1'S,4S)-6'-bromo-4-methoxy-4''-methyl-3'H-dispiro[cyclohexane-1,2'-indene-1',2''-imidazol-5''-yl]-4-methoxy-4''-methyl-3'H-dispiro[cyclohexane-1,2'-indene-1',2''-imidazol]-5''-imine (1) Geometric parameters (Å, º)

**Table:**

Br1—C7	1.9071 (16)	C19—C20	1.4925 (19)
Br2—C25	1.9005 (15)	C19—N1	1.3616 (18)
C1—C2	1.4940 (19)	C19—N4	1.3115 (18)
C1—N1	1.3327 (19)	C20—C21	1.4917 (19)
C1—N2	1.3250 (19)	C20—N5	1.2809 (19)
C2—C3	1.485 (2)	C21—H21A	0.9800
C2—N3	1.2818 (19)	C21—H21B	0.9800
C3—H3A	0.9800	C21—H21C	0.9800
C3—H3B	0.9800	C22—C23	1.5169 (19)
C3—H3C	0.9800	C22—C30	1.5775 (19)
C4—C5	1.514 (2)	C22—N4	1.4513 (17)
C4—C12	1.5697 (19)	C22—N5	1.4816 (17)
C4—N2	1.4471 (18)	C23—C24	1.383 (2)
C4—N3	1.4770 (17)	C23—C28	1.392 (2)
C5—C6	1.379 (2)	C24—H24	0.9500
C5—C10	1.390 (2)	C24—C25	1.389 (2)
C6—H6	0.9500	C25—C26	1.386 (2)
C6—C7	1.387 (2)	C26—H26	0.9500
C7—C8	1.389 (2)	C26—C27	1.387 (2)
C8—H8	0.9500	C27—H27	0.9500
C8—C9	1.393 (2)	C27—C28	1.390 (2)
C9—H9	0.9500	C28—C29	1.504 (2)
C9—C10	1.392 (2)	C29—H29A	0.9900
C10—C11	1.512 (2)	C29—H29B	0.9900
C11—H11A	0.9900	C29—C30	1.5489 (19)
C11—H11B	0.9900	C30—C31	1.5271 (19)
C11—C12	1.556 (2)	C30—C35	1.5441 (19)
C12—C13	1.530 (2)	C31—H31A	0.9900
C12—C17	1.545 (2)	C31—H31B	0.9900
C13—H13A	0.9900	C31—C32	1.534 (2)
C13—H13B	0.9900	C32—H32A	0.9900
C13—C14	1.528 (2)	C32—H32B	0.9900
C14—H14A	0.9900	C32—C33	1.527 (2)
C14—H14B	0.9900	C33—H33	1.0000
C14—C15	1.516 (2)	C33—C34	1.519 (2)
C15—H15	1.0000	C33—O2	1.4284 (19)
C15—C16	1.522 (2)	C34—H34A	0.9900
C15—O1	1.4311 (19)	C34—H34B	0.9900
C16—H16A	0.9900	C34—C35	1.531 (2)
C16—H16B	0.9900	C35—H35A	0.9900
C16—C17	1.529 (2)	C35—H35B	0.9900
C17—H17A	0.9900	C36—H36A	0.9800
C17—H17B	0.9900	C36—H36B	0.9800
C18—H18A	0.9800	C36—H36C	0.9800
C18—H18B	0.9800	C36—O2	1.412 (2)
C18—H18C	0.9800	N2—H2	0.823 (14)
C18—O1	1.421 (2)		
			
N1—C1—C2	125.17 (13)	N5—C20—C21	125.43 (13)
N2—C1—C2	105.82 (12)	C20—C21—H21A	109.5
N2—C1—N1	128.98 (13)	C20—C21—H21B	109.5
C3—C2—C1	123.12 (13)	C20—C21—H21C	109.5
N3—C2—C1	111.32 (12)	H21A—C21—H21B	109.5
N3—C2—C3	125.56 (13)	H21A—C21—H21C	109.5
C2—C3—H3A	109.5	H21B—C21—H21C	109.5
C2—C3—H3B	109.5	C23—C22—C30	102.04 (11)
C2—C3—H3C	109.5	N4—C22—C23	114.04 (12)
H3A—C3—H3B	109.5	N4—C22—C30	112.98 (11)
H3A—C3—H3C	109.5	N4—C22—N5	106.87 (11)
H3B—C3—H3C	109.5	N5—C22—C23	109.73 (11)
C5—C4—C12	102.45 (11)	N5—C22—C30	111.22 (11)
N2—C4—C5	114.25 (12)	C24—C23—C22	128.04 (13)
N2—C4—C12	114.48 (12)	C24—C23—C28	122.24 (13)
N2—C4—N3	105.19 (11)	C28—C23—C22	109.71 (12)
N3—C4—C5	109.74 (11)	C23—C24—H24	121.6
N3—C4—C12	110.81 (11)	C23—C24—C25	116.76 (14)
C6—C5—C4	127.50 (13)	C25—C24—H24	121.6
C6—C5—C10	122.50 (14)	C24—C25—Br2	118.99 (12)
C10—C5—C4	109.99 (13)	C26—C25—Br2	118.59 (12)
C5—C6—H6	121.3	C26—C25—C24	122.42 (14)
C5—C6—C7	117.34 (14)	C25—C26—H26	120.2
C7—C6—H6	121.3	C25—C26—C27	119.68 (14)
C6—C7—Br1	118.08 (12)	C27—C26—H26	120.2
C6—C7—C8	121.94 (15)	C26—C27—H27	120.4
C8—C7—Br1	119.97 (12)	C26—C27—C28	119.21 (14)
C7—C8—H8	120.3	C28—C27—H27	120.4
C7—C8—C9	119.48 (14)	C23—C28—C29	110.20 (12)
C9—C8—H8	120.3	C27—C28—C23	119.68 (14)
C8—C9—H9	120.2	C27—C28—C29	130.11 (13)
C10—C9—C8	119.57 (14)	C28—C29—H29A	111.1
C10—C9—H9	120.2	C28—C29—H29B	111.1
C5—C10—C9	119.10 (14)	C28—C29—C30	103.23 (11)
C5—C10—C11	109.99 (13)	H29A—C29—H29B	109.1
C9—C10—C11	130.91 (14)	C30—C29—H29A	111.1
C10—C11—H11A	111.0	C30—C29—H29B	111.1
C10—C11—H11B	111.0	C29—C30—C22	101.61 (11)
C10—C11—C12	103.59 (11)	C31—C30—C22	112.81 (11)
H11A—C11—H11B	109.0	C31—C30—C29	113.80 (12)
C12—C11—H11A	111.0	C31—C30—C35	109.08 (12)
C12—C11—H11B	111.0	C35—C30—C22	109.10 (11)
C11—C12—C4	101.86 (11)	C35—C30—C29	110.20 (11)
C13—C12—C4	110.97 (12)	C30—C31—H31A	109.3
C13—C12—C11	114.79 (12)	C30—C31—H31B	109.3
C13—C12—C17	109.50 (12)	C30—C31—C32	111.72 (12)
C17—C12—C4	109.15 (11)	H31A—C31—H31B	107.9
C17—C12—C11	110.28 (12)	C32—C31—H31A	109.3
C12—C13—H13A	108.9	C32—C31—H31B	109.3
C12—C13—H13B	108.9	C31—C32—H32A	109.3
H13A—C13—H13B	107.7	C31—C32—H32B	109.3
C14—C13—C12	113.34 (12)	H32A—C32—H32B	108.0
C14—C13—H13A	108.9	C33—C32—C31	111.61 (13)
C14—C13—H13B	108.9	C33—C32—H32A	109.3
C13—C14—H14A	109.3	C33—C32—H32B	109.3
C13—C14—H14B	109.3	C32—C33—H33	109.0
H14A—C14—H14B	107.9	C34—C33—C32	111.30 (12)
C15—C14—C13	111.74 (13)	C34—C33—H33	109.0
C15—C14—H14A	109.3	O2—C33—C32	112.32 (13)
C15—C14—H14B	109.3	O2—C33—H33	109.0
C14—C15—H15	109.0	O2—C33—C34	106.11 (13)
C14—C15—C16	109.82 (13)	C33—C34—H34A	109.5
C16—C15—H15	109.0	C33—C34—H34B	109.5
O1—C15—C14	106.87 (13)	C33—C34—C35	110.78 (13)
O1—C15—H15	109.0	H34A—C34—H34B	108.1
O1—C15—C16	113.19 (13)	C35—C34—H34A	109.5
C15—C16—H16A	109.7	C35—C34—H34B	109.5
C15—C16—H16B	109.7	C30—C35—H35A	109.1
C15—C16—C17	109.84 (12)	C30—C35—H35B	109.1
H16A—C16—H16B	108.2	C34—C35—C30	112.68 (12)
C17—C16—H16A	109.7	C34—C35—H35A	109.1
C17—C16—H16B	109.7	C34—C35—H35B	109.1
C12—C17—H17A	109.0	H35A—C35—H35B	107.8
C12—C17—H17B	109.0	H36A—C36—H36B	109.5
C16—C17—C12	112.81 (12)	H36A—C36—H36C	109.5
C16—C17—H17A	109.0	H36B—C36—H36C	109.5
C16—C17—H17B	109.0	O2—C36—H36A	109.5
H17A—C17—H17B	107.8	O2—C36—H36B	109.5
H18A—C18—H18B	109.5	O2—C36—H36C	109.5
H18A—C18—H18C	109.5	C1—N1—C19	114.92 (12)
H18B—C18—H18C	109.5	C1—N2—C4	110.02 (12)
O1—C18—H18A	109.5	C1—N2—H2	119.8 (12)
O1—C18—H18B	109.5	C4—N2—H2	130.1 (12)
O1—C18—H18C	109.5	C2—N3—C4	107.61 (11)
N1—C19—C20	123.54 (12)	C19—N4—C22	107.66 (11)
N4—C19—C20	108.12 (12)	C20—N5—C22	106.51 (11)
N4—C19—N1	128.29 (13)	C18—O1—C15	113.31 (14)
C21—C20—C19	123.79 (12)	C36—O2—C33	113.60 (15)
N5—C20—C19	110.78 (12)		
			
Br1—C7—C8—C9	178.42 (11)	C24—C23—C28—C29	178.36 (13)
Br2—C25—C26—C27	179.54 (12)	C24—C25—C26—C27	−0.7 (2)
C1—C2—N3—C4	1.52 (16)	C25—C26—C27—C28	0.2 (2)
C2—C1—N1—C19	−174.58 (13)	C26—C27—C28—C23	0.4 (2)
C2—C1—N2—C4	1.35 (16)	C26—C27—C28—C29	−178.16 (15)
C3—C2—N3—C4	−177.80 (14)	C27—C28—C29—C30	157.17 (15)
C4—C5—C6—C7	−177.07 (13)	C28—C23—C24—C25	−0.1 (2)
C4—C5—C10—C9	176.23 (13)	C28—C29—C30—C22	33.89 (13)
C4—C5—C10—C11	−3.85 (16)	C28—C29—C30—C31	155.44 (12)
C4—C12—C13—C14	−171.17 (13)	C28—C29—C30—C35	−81.68 (14)
C4—C12—C17—C16	174.63 (12)	C29—C30—C31—C32	68.08 (16)
C5—C4—C12—C11	−33.48 (13)	C29—C30—C35—C34	−69.81 (15)
C5—C4—C12—C13	−156.11 (12)	C30—C22—C23—C24	−156.70 (14)
C5—C4—C12—C17	83.12 (13)	C30—C22—C23—C28	22.73 (14)
C5—C4—N2—C1	119.89 (13)	C30—C22—N4—C19	−120.81 (13)
C5—C4—N3—C2	−124.03 (13)	C30—C22—N5—C20	123.30 (12)
C5—C6—C7—Br1	−179.74 (10)	C30—C31—C32—C33	56.31 (17)
C5—C6—C7—C8	0.6 (2)	C31—C30—C35—C34	55.79 (16)
C5—C10—C11—C12	−18.25 (16)	C31—C32—C33—C34	−55.19 (17)
C6—C5—C10—C9	−2.8 (2)	C31—C32—C33—O2	−174.01 (12)
C6—C5—C10—C11	177.14 (13)	C32—C33—C34—C35	54.55 (17)
C6—C7—C8—C9	−2.0 (2)	C32—C33—O2—C36	−74.54 (18)
C7—C8—C9—C10	0.9 (2)	C33—C34—C35—C30	−55.86 (16)
C8—C9—C10—C5	1.4 (2)	C34—C33—O2—C36	163.64 (15)
C8—C9—C10—C11	−178.53 (15)	C35—C30—C31—C32	−55.40 (16)
C9—C10—C11—C12	161.66 (15)	N1—C1—C2—C3	−4.1 (2)
C10—C5—C6—C7	1.8 (2)	N1—C1—C2—N3	176.53 (14)
C10—C11—C12—C4	31.57 (14)	N1—C1—N2—C4	−176.97 (14)
C10—C11—C12—C13	151.55 (12)	N1—C19—C20—C21	4.0 (2)
C10—C11—C12—C17	−84.21 (14)	N1—C19—C20—N5	−175.33 (13)
C11—C12—C13—C14	74.04 (17)	N1—C19—N4—C22	175.04 (14)
C11—C12—C17—C16	−74.26 (15)	N2—C1—C2—C3	177.46 (14)
C12—C4—C5—C6	−157.02 (14)	N2—C1—C2—N3	−1.88 (17)
C12—C4—C5—C10	24.03 (15)	N2—C1—N1—C19	3.4 (2)
C12—C4—N2—C1	−122.41 (13)	N2—C4—C5—C6	−32.6 (2)
C12—C4—N3—C2	123.55 (13)	N2—C4—C5—C10	148.42 (12)
C12—C13—C14—C15	54.37 (18)	N2—C4—C12—C11	−157.73 (12)
C13—C12—C17—C16	52.97 (16)	N2—C4—C12—C13	79.64 (15)
C13—C14—C15—C16	−57.49 (18)	N2—C4—C12—C17	−41.12 (16)
C13—C14—C15—O1	179.37 (13)	N2—C4—N3—C2	−0.68 (15)
C14—C15—C16—C17	59.04 (17)	N3—C4—C5—C6	85.21 (17)
C14—C15—O1—C18	−176.19 (14)	N3—C4—C5—C10	−93.74 (14)
C15—C16—C17—C12	−58.08 (17)	N3—C4—C12—C11	83.52 (13)
C16—C15—O1—C18	62.79 (19)	N3—C4—C12—C13	−39.11 (16)
C17—C12—C13—C14	−50.61 (17)	N3—C4—C12—C17	−159.88 (11)
C19—C20—N5—C22	−0.97 (15)	N3—C4—N2—C1	−0.52 (15)
C20—C19—N1—C1	171.64 (13)	N4—C19—C20—C21	−178.47 (13)
C20—C19—N4—C22	−2.37 (15)	N4—C19—C20—N5	2.22 (17)
C21—C20—N5—C22	179.73 (13)	N4—C19—N1—C1	−5.4 (2)
C22—C23—C24—C25	179.31 (13)	N4—C22—C23—C24	−34.6 (2)
C22—C23—C28—C27	−179.95 (13)	N4—C22—C23—C28	144.88 (12)
C22—C23—C28—C29	−1.11 (16)	N4—C22—C30—C29	−157.12 (11)
C22—C30—C31—C32	−176.82 (11)	N4—C22—C30—C31	80.65 (15)
C22—C30—C35—C34	179.43 (12)	N4—C22—C30—C35	−40.75 (15)
C23—C22—C30—C29	−34.24 (13)	N4—C22—N5—C20	−0.44 (15)
C23—C22—C30—C31	−156.47 (11)	N5—C22—C23—C24	85.28 (17)
C23—C22—C30—C35	82.13 (13)	N5—C22—C23—C28	−95.29 (14)
C23—C22—N4—C19	123.27 (13)	N5—C22—C30—C29	82.70 (13)
C23—C22—N5—C20	−124.56 (13)	N5—C22—C30—C31	−39.53 (15)
C23—C24—C25—Br2	−179.61 (10)	N5—C22—C30—C35	−160.93 (11)
C23—C24—C25—C26	0.7 (2)	N5—C22—N4—C19	1.84 (15)
C23—C28—C29—C30	−21.51 (15)	O1—C15—C16—C17	178.38 (13)
C24—C23—C28—C27	−0.5 (2)	O2—C33—C34—C35	177.03 (12)

### (R,S)/(S,R)-(1r,1'S,4S,E)-6'-Bromo-N-[(1r,1'S,4S)-6'-bromo-4-methoxy-4''-methyl-3'H-dispiro[cyclohexane-1,2'-indene-1',2''-imidazol-5''-yl]-4-methoxy-4''-methyl-3'H-dispiro[cyclohexane-1,2'-indene-1',2''-imidazol]-5''-imine (1) Hydrogen-bond geometry (Å, º)

**Table:**

*D*—H···*A*	*D*—H	H···*A*	*D*···*A*	*D*—H···*A*
N2—H2···N4	0.82 (1)	1.95 (2)	2.5549 (16)	129 (2)
